# Novel Insights into Anthocyanin Synthesis in the Calyx of Roselle Using Integrated Transcriptomic and Metabolomic Analyses

**DOI:** 10.3390/ijms232213908

**Published:** 2022-11-11

**Authors:** Jing Li, Yunqing Li, Mei Li, Lihui Lin, Jianmin Qi, Jiantang Xu, Liwu Zhang, Pingping Fang, Aifen Tao

**Affiliations:** 1 Key Laboratory of Ministry of Education for Genetics, Breeding and Multiple Utilization of Crops, Fujian Agriculture and Forestry University, Fuzhou 350002, China; 2 Fujian Key Laboratory of Crop Breeding for Design, Fujian Agriculture and Forestry University, Fuzhou 350002, China

**Keywords:** roselle, anthocyanin, transcriptome, metabolome, synthesis mechanism

## Abstract

Roselle (*Hibiscus sabdariffa* L.) is an annual herbaceous plant of the genus *Hibiscus* in family Malvaceae. Roselle calyxes are rich in anthocyanins, which play important roles in human health. However, limited information is available on anthocyanin biosynthesis in the roselle calyx. In this study, transcriptomic and metabolomic analyses were performed to identify the key genes involved in anthocyanin biosynthesis in the roselle calyx. Three roselle cultivars with different calyx colors, including FZ-72 (red calyx, R), Baitao K (green calyx, G), and MG5 (stripped calyx, S), were used for metabolomic analyses with UPLC-Q-TOF/MS and RNA-seq. Forty-one compounds were quantified, including six flavonoids and 35 anthocyanins. The calyx of FZ-72 (red calyx) had the highest contents of anthocyanin derivatives such as delphinidin-3-O-sambubioside (955.11 μg/g) and cyanidin-3-O-sambubioside (531.37 μg/g), which were responsible for calyx color, followed by those in MG5 (stripped calyx) (851.97 and 330.06 μg/g, respectively). Baitao K (green calyx) had the lowest levels of these compounds. Furthermore, RNA-seq analysis revealed 114,415 differentially expressed genes (DEGs) in the calyxes at 30 days after flowering (DAF) for the corresponding cultivars FZ-72 (R), Baitao K (G), and MG5(S). The gene expression levels in the calyxes of the three cultivars were compared at different flowering stages, revealing 11,555, 11,949, and 7177 DEGs in R vs. G, R vs. S, and G vs. S, respectively. Phenylpropanoid and flavonoid biosynthesis pathways were found to be enriched. In the flavonoid pathway, 29, 28, and 27 genes were identified in G vs. R, G vs. S, and S vs. R, respectively. In the anthocyanin synthesis pathway, two, two, and one differential genes were identified in the three combinations; these differential genes belonged to the *UFGT* gene family. After joint analysis of the anthocyanin content in roselle calyxes, nine key genes belonging to the *CHS*, *CHI*, *UFGT*, *FLS*, *ANR*, *DFR*, *CCoAOMT*, *SAT*, and *HST* gene families were identified as strongly related to anthocyanin synthesis. These nine genes were verified using qRT-PCR, and the results were consistent with the transcriptome data. Overall, this study presents the first report on anthocyanin biosynthesis in roselle, laying a foundation for breeding roselle cultivars with high anthocyanin content.

## 1. Introduction

Roselle (*Hibiscus sabdariffa* L.), a highly self-pollinated member of the Malvaceae family, is an essential annual crop in the tropics and subtropics [1]. It has high nutritional, medicinal, and economic values. Roselle plants are a popular source of vegetable protein, fat, and minerals [2]. Roselle calyx is the most profitable part [3]; it is consumed as jam, syrup, and hot or cold drinks, and used as a natural food colorant [4]. Roselle calyx extracts demonstrate various beneficial properties, including antioxidant, anticancer, antihypertensive, hypolipidemic, hepatoprotective, anti-stress, antispasmodic, diuretic, and antidiarrheal activities [5]. In addition to being used as food, various parts of roselle plants have been utilized in traditional medicine for preventing diseases such as diabetes, cancer, hypertension [6], and obesity [6,7]. Furthermore, roselle can be used commercially as an alternative fiber source [8]. Overall, roselle is widely used in the food, beverage, and pharmaceutical industries because of its high vitamin C and anthocyanin contents [9].

Flavonoids are essential phenolic compounds and include anthocyanins, flavones, and proanthocyanidins, which are widely present in plant leaves and fruits. Anthocyanins are naturally occurring polyphenols responsible for the colors of most flowers and fruits [10]. They are mainly orange, red, purple, and blue, whereas flavonoids and flavonols are mainly yellow to white [11]. Thus, anthocyanins help reduce the harmful effects of reactive oxygen species during unfavorable environmental conditions [12]. The anthocyanin content in roselle calyxes increases markedly under salt and drought stress [6,7]. Anthocyanins and flavonoids have recently attracted considerable attention for their anticancer, antioxidant, and anti-inflammatory properties [13]. To date, more than 702 anthocyanins have been isolated from plants [14], including cyanidin, delphinidin, malvidin, pelargonidin, peonidin, and petunidin [15].

Anthocyanin biosynthesis has been well-characterized in many plant species, including *Camellia sinensis* [16], *Gossypium* spp. [17], *Solanum lycopersicum* [18], *Malus pumila Mill*, and *Pyrus* spp. [19]. Anthocyanin metabolic pathways in different crops have individual differences, which result in different plant colors, such as purple, blue, and red. This phenomenon is attributed to differences in the expression of critical genes and enzymes in the biosynthetic pathway, including *CHS*, *ANS*, MYB, DFR, bHLH, and WD40. The *CHS* and *F3H* genes of roselle have been cloned and structurally analyzed using homologous cloning. The essential genes involved in anthocyanin biosynthesis have also been studied in kenaf, a species related to roselle; eight structural gene types and three transcription factor families have been found to play vital roles in anthocyanin synthesis [20]. The sequence of amino acids in the substrate-specific binding region is known to determine the specificity of *DFR* for different substrate binding, ultimately leading to anthocyanin synthesis and phenotype color [21]. Upon comparing purple pepper with green pepper, researchers found that early biosynthesis genes (*CHS*, *CHI*, *F3H*) did not cause anthocyanin synthesis [22]. Preliminary analysis of gene functions related to procyanidin synthesis and regulation in colored cotton revealed that five essential genes (*GhCHS*, *GhF3’H*, *GhF3’5’H*, *GhLAR*, and *GhANR*) showed similar expression patterns in anthocyanin synthesis in colored cotton [23].

Roselle is a rich source of anthocyanins [24]. However, few studies have examined the biosynthesis of anthocyanins in roselles. This study aimed to identify the anthocyanin biosynthesis genes in roselle by comparing the gene expression levels in three roselle cultivars with different calyx colors using ultra-performance liquid chromatography UPLC-Q-TOF/MS combined with RNA-seq. This study provides a basis for screening roselle cultivars with high anthocyanin content and provides a theoretical basis for molecular marker development and gene cloning in future.

## 2. Results

**Figure 1:**
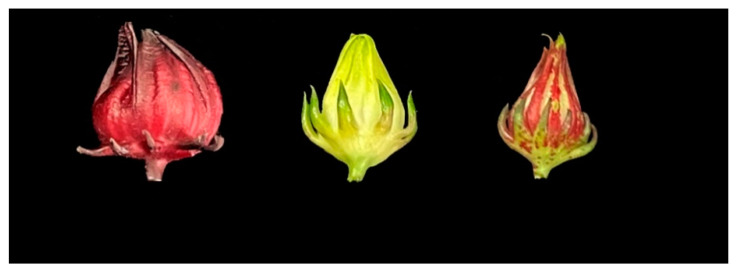
Represent images of calyx from the three roselle cultivars: FZ-72 (**the left**), Baitao K (**the middle**) and MG5 (**the right**).

For the purposes of the present study, we characterized and compared both the anthocynin metabolites and transcriptional activity of three roselle cultivars (FZ-72, Baitao K and MG5) with different calyx colors (Figure 1). The calyxes of FZ-72 are dark red, whereas which of Baitao K are green, and MG5 has stripped calyxes.

### 2.1. Anthocyanin Content in Roselle Calyxes

**Figure 2:**
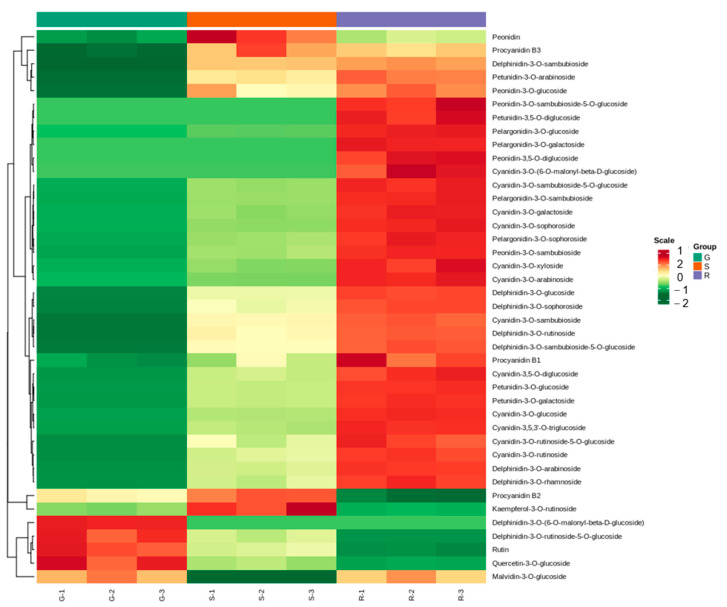
Heat map showing the contents of 41 flavonoids and anthocyanins in different cultivars. The abscissa is the three repeats of different roselle cultivars, and the ordinate indicates the types of anthocyanins and flavonoids. Red color represents high content, whereas green represents low content; yellow color indicates intermediate content. R, FZ-72 (red calyx); G, Baitao K (green calyx); S, MG5 (stripped calyx).

We hypothesized that anthocyanins are responsible for the different colors in roselle calyxes. To examine the primary metabolites of roselle, the calyxes of three different roselle cultivars were collected for observation at 30 d after full bloom (Figure 1) using UPLC-Q-TOF/MS (Table 1). In total, 108 metabolites (Supplement Table S1) were measured, of which 41 were obtained from roselle sepals, including 12 cyanidins, nine delphinidins, one malvidin, four pelargonidins, five peonidins, four petunidins, three procyanidins, and three flavonoids (Figure 2). The heat map showed diametrically opposite trends in the metabolite contents of cultivars R and G. Furthermore, the top three anthocyanins based on content in the calyxes of R were dephinidin-3-O-sambubioside (955.11 μg/g), cyanidin-3-O-sambubioside (531.37 μg/g), and delphinidin-3-O-sophoroside (85.08 μg/g). In addition to delphinidin-3-O-sambubioside and cyanidin-3-O-sambubioside, rutin was among the top three anthocyanins in S cultivars. However, in cultivar S flavonoids showed higher content whereas the other anthocyanins were present at less than 1 μg/g.

### 2.2. Sample Correlation Analysis of Roselle Calyxes and Principal Component Analysis

**Figure 3:**
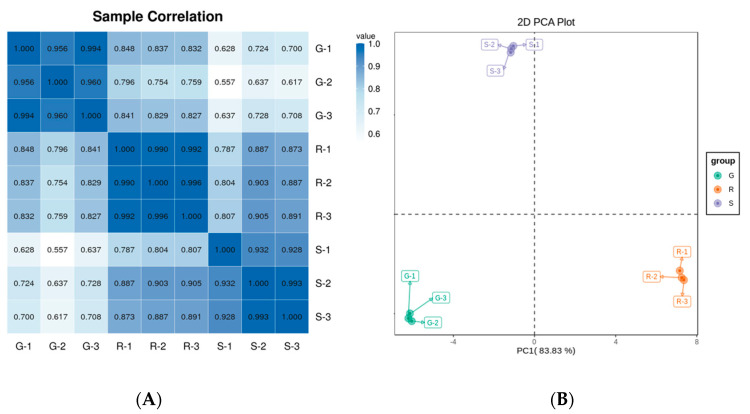
Sample correlation analysis and principal component analysis of sepals in different roselle cultivars. (**A**) Sample correlation analysis: Closeness of the correlation coefficient to 1 indicates the correlation strength of the cultivars. (**B**) The three roselle cultivars were separated from each other and the biological repeats were clustered together.

To determine the variability of different varieties of roselle metabolites and the consistency between replicates, we performed principal component analysis (PCA) and sample correlation analysis. A heatmap of the associations between samples indicated the reliability of biological replicates for the three cultivars (Figure 3A). Pearson correlation coefficients between samples were visualized in the form of heat maps to display the correlations between samples. The scatter points corresponding to the samples of the three groups were clustered together within a group. Repeatability within the group was relatively sound with a reasonable degree of discrimination between groups. The correlation coefficients for all three biological replicates were greater than 0.9. In the meantime, the metabolites were separated into three groups based on PCA (Figure 3B). The first principal component (PC1) explained 83.3% of the total variance, and that cultivar R (FZ-72, red calyx) was separated from G (Baitao K, green calyx) and S (MG5, stripped calyx). The second principal component (PC2) explained 15.49% of the variance, and cultivar S was separated from cultivars G and R. The results showed apparent differences in the anthocyanin content and types among the three cultivars, with those in S being significantly different from those in G and R. These results demonstrated good biological repeatability of different roselle cultivars and the reliability of the results.

### 2.3. Differential Metabolite of Roselle

In order to obtain differentially expressed metabolites (DEMs) among three roselle cultivars, roselle metabolites with fold change ≥2 and fold change ≤0.5 were selected for further analysis. In addition, Kyoto Encyclopedia of Genes and Genomes (KEGG) was used for the functional annotation and enrichment analysis of DEMs. Thirty-seven metabolites were screened from the G and R cultivars, among which six DEMs (cyanidin-3-O-sambubioside, delphinidin-3-O-sambubioside, cyanidin-3-O-glucoside, delphinidin-3-O-rutinoside, procyanidin B3, and peonidin) were significantly up-regulated and two (rutin and quercetin-3-O-glucoside) were significantly down-regulated (Supplement Figure S1A). In total, 33 metabolites were screened from G and S; among these, seven DEMs (delphinidin-3-O-sambubioside, cyanidin-3-O-sambubioside, peonidin, delphinidin-3-O-rutinoside, cyanidin-3-O-glucoside, procyanidinB3, kaempferol-3-O-rutinoside) were significantly up-regulated and two (delphinidin-3-O-rutinoside-5-O-glucoside, rutin) were significantly down-regulated (Supplement Figure S1B). Further, 30 metabolites with significant differences were screened from S and R, among which 15 were significantly up-regulated and five were significantly down-regulated, which met the screening conditions (Supplement Figure S1C). The DEMs were classified into four categories: anthocyanin biosynthesis (ko00942), metabolic pathways (ko01100), biosynthesis of secondary metabolites (ko01110), and flavone and flavonol biosynthesis (ko00944). In total, 37, 33, and 30 DEMs were screened from G and R, G and S, and S and R, respectively. Among these combinations, 21, 19, and 12 DEMS were annotated into the anthocyanin synthesis pathway, accounting for 90.48%, 90.48%, and 80%, respectively. Metabolomic analysis of two cultivars with significant differences indicated that the expression of delphinidin-3-O-sambubioside in cultivar R was 3537-fold than that in cultivar G and the content of cyanidin-3-O-sambubioside in cultivar R was 4830-fold than that in cultivar G. Cyanidin-3-O-sambubioside and delphinidin-3-O-sambubioside play essential roles in roselle anthocyanin biosynthesis.

### 2.4. Roselle Transcriptome and De Novo Assembly

In our experiment, transcriptome sequencing (RNA-seq) was performed on the Illumina HiSeq^TM^2500 platform to clarify the reasons for the differential accumulation of anthocyanins in different roselle cultivars. Here, 114,415 unigenes with an average length of 780 bp were obtained from the three cultivars. The average GC content of the unigenes was 40.46%. The length and quantity of N50 unigenes were 19,260 and 1325, respectively, showing relatively stable quality. Using Benchmarking Universal Single-Copy Ortholog (BUSCO) evaluation, 1077 genes complete BUSCOs and 977 genes of complete and single-copy BUSCOs were screened. Further, 80 genes of complete and duplicated BUSCOs, 207 genes of fragmented BUSCOs, and 156 genes of missing BUSCOs were identified.

**Figure 4:**
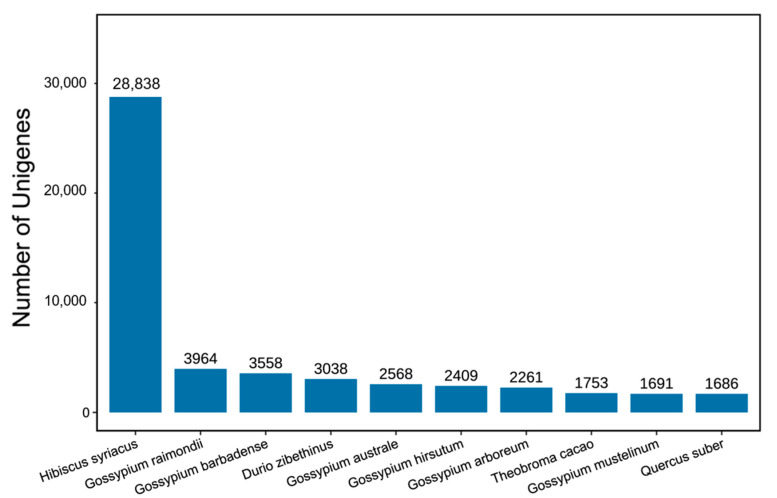
Alignment of homologous sequences from different species. The “X” axis shows different species, and the “Y” axis indicates the number of similar unigenes.

In total, 65,762 unigenes obtained from the assembly were annotated in the NR database. The homologous sequences of the species were determined by comparing the unigene sequences with the NR database. The number of homologous sequences for each species was statistically compared and 28,838 genes were found to be homologous to those of *Hibiscus syriacus* (Figure 4). Homologous sequences aligned with *Hibiscus* accounted for 43% of the unigenes annotated to the NR database, indicating the most aligned species. Homologous gene sequence alignment results indicated that roselle and *Hibiscus* were more closely related. Using KOG database annotations, unigenes were divided into 25 functional partitions (Supplement Figure S2). The first five categories were general function prediction only, signal transduction mechanisms, post-translational modification, protein turnover, chaperones, translation, ribosomal structure and biogenesis, intracellular trafficking, secretion, and vesicular transport. Further, 1490 genes were annotated for secondary metabolite biosynthesis, transport, and catabolism classification. Gene ontology (GO) analysis divided these roselle unigenes into the categories of molecular function, cellular component, and biological processes. The proportion of unigenes distributed in biological processes was relatively high. Further, 63,073 assembled unigenes were annotated in the KEGG database, accounting for approximately 55% of the total. KEGG analysis divided the unigenes of roselle into five modules: metabolism, genetic information processing, environmental information processing, organismal systems, and cellular processes. Moreover, most unigenes were annotated as metabolic pathways.

### 2.5. Differentially Expressed Genes (DEGs)

**Figure 5:**
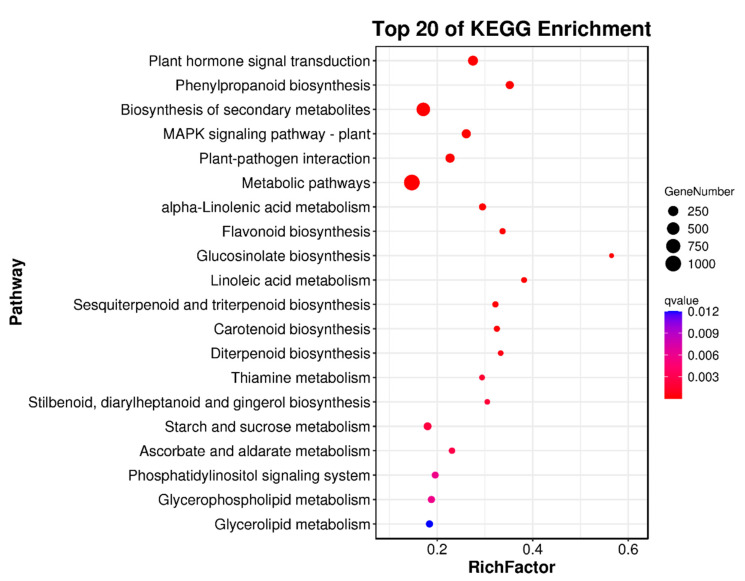
Bubble plot of differential genes enrichment for G vs R cultivars. The top 20 pathways with the smallest Q values were used to construct the graph. KEGG enrichment of DEGs among different roselle cultivars. The “X” axis shows the enrichment score; the “Y” axis shows the number of DEGs mapped to each KEGG pathway.

Transcriptome analysis was conducted to screen the differentially expressed genes (DEGs) among the three samples, and the result showed that 4903 genes were significantly up-regulated, whereas 6652 genes were significantly down-regulated in cultivar R compared with those in G. Among the DEGs between G and S, 5092 were up-regulated, and 6857 were down-regulated significantly. Compared with cultivar R, 3899 DEGs were up-regulated and 3278 were significantly down-regulated in S cultivars. Volcano plot analysis based on significantly different genes in each comparison group (Supplement Figure S3) indicated many differential genes between different comparison groups, among which cultivars S and R showed the smallest difference in the number of DEGs. However, KEGG analysis showed that few genes differed in the anthocyanin metabolic pathways among the three cultivars. The genes between cultivars G and R were mainly involved in the biosynthesis of secondary metabolites, metabolic pathways, and plant hormone signal transduction (Figure 5). Among these, metabolic pathways accounted for the most significant proportion. KEGG enrichment analysis of G and S cultivars showed that the differential genes were mainly enriched in the biosynthesis of secondary metabolites and metabolic pathways (Supplement Figure S4A), and that the distribution proportion of differential genes in the KEGG pathway between S and G was consistent with that of G and R (Supplement Figure S4B).

As anthocyanins are biological flavonoids, the flavonoid synthesis pathway (ko00941) and anthocyanin synthesis pathway (ko00942) under secondary metabolite pathways were chosen for further study. The flavonoid biosynthesis pathway between R and G involved 29 genes, including *CHS*, *CHI, FLS*, *DFR*, *ANS*, and *ANR*. Moreover, two genes (*UFGT*) were involved in the anthocyanin synthesis pathway. We further identified 28 genes involved in the flavonoid synthesis pathway between G and S, including *CHS*, *CHI*, *SAT*, *HST*, *ANR*, and *SAT*. Further, there were two different genes (*UFGT*) in this comparison. Twenty-six DEGs were found in the flavonoid biosynthesis pathway between S and R, and only one DEG found in the anthocyanin biosynthesis pathway. In total, 49 significantly different unigenes were screened in the three combinations of the flavonoid synthesis pathway (ko00941) and the anthocyanin synthesis pathway (ko00942) (Supplement Table S2). There were four DEGs in the three combinations (Supplement Figure S5), three (Unigene0008117, Unigene0074947, and Unigene0008826) in the flavonoid synthesis pathway (ko00941), and one (Unigene0030931) in the anthocyanin synthesis pathway (ko00942).

### 2.6. Transcription Factor

**Figure 6:**
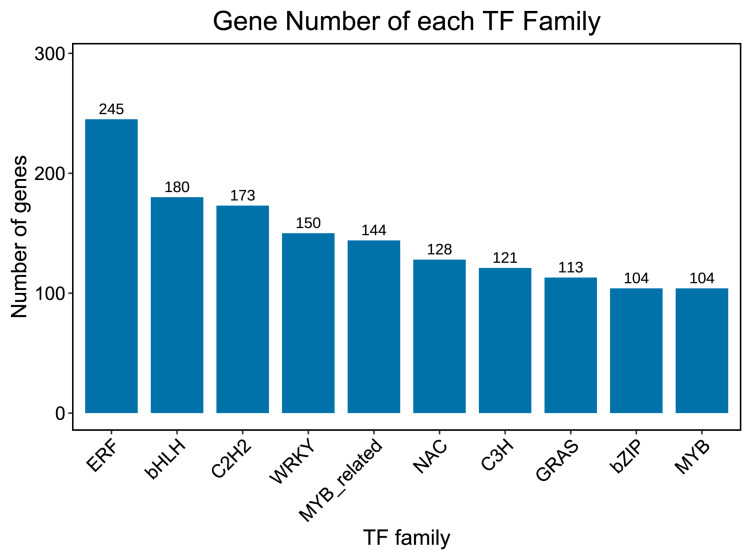
Transcription factor statistics. The “X” axis shows the top 10 transcription factor families, and the “Y” axis represents the number of genes.

To identify the transcription factors (TF) in roselle, the predicted protein sequences were compared using the TF database HMMSCAN. According to TF-related annotation analysis, the top 10 transcription factor families were ERF, bHLH, C2H2, WRKY, MYB-related, NAC, C3H, GASE, bZIP, and MYB. Among these, ERF accounted for the highest proportion (Figure 6). The transcription factor MYB forms a WD40 protein complex with bHLH, thereby participating in anthocyanin synthesis. Seventy MYB and 94 bHLH transcription factors were screened through joint analysis, and their interactions were studied further. In the Chinese rose [25], anthocyanin synthesis is closely related to the WD40 protein complex formed by MYB and bHLH. The R2R3-MYB protein can activate the structure of anthocyanins and promote anthocyanin synthesis and accumulation.

### 2.7. Conjoint Analysis

**Figure 7:**
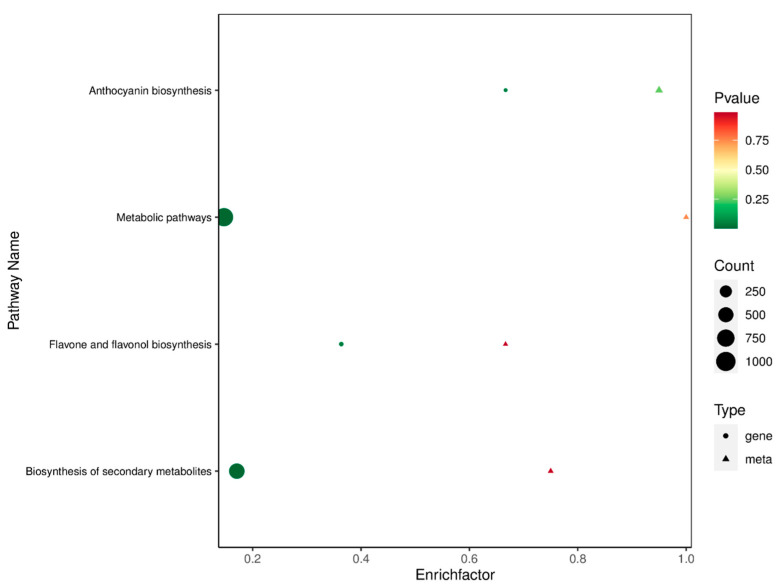
Bubble diagram for the combined analysis of differential genes and metabolites between G (Baitao K, green calyx) and R (FZ-72, red calyx).

In order to verify the genes in the metabolic pathways associated with anthocyanin synthesis in roselle, Pearson correlation analysis was used to combine the RNA-seq data of different roselle calyxes and the LC-MS/MS results. Furthermore, the differential genes obtained using transcriptome and metabolome analyses were annotated using KEGG pathways. In this case, we found that the associated genes were mainly distributed in ko001100, ko00942, ko00944, and ko00941 (Figure 7, supplement Figure S6A,B). Combined analysis of 41 anthocyanins detected in roselle calyxes and unigenes assembled using the transcriptomes showed that 748, 959, and 433 genes strongly associated with anthocyanin synthesis were screened in the combinations G vs. R, G vs. S, and S vs. R, respectively. In the comprehensive analysis of the three cultivars, 1215 screened unigenes were found to play an essential role in roselle anthocyanin synthesis.

The flavonoid (ko00941) and anthocyanin (ko00942) synthesis pathways were selected for further analysis. Twenty-three essential genes were screened in the combination of G and R cultivars, which is the highest number associated with the most significant phenotypic differences. The combined analysis also showed that 23 DEGs were significantly associated with seven anthocyanins. Combined analysis of G and S cultivars confirmed that the synthesis of six DEMs was significantly associated with 16 DEGs. Among these, 13 DEGs were involved in the synthesis and transport of all DEMs. However, *ANR* and *FAOMT* were not associated with cyanidin-3-O-glucoside. A certain similarity is speculated in the expression of these two genes involved in anthocyanin synthesis in roselle calyxes. *CHI3*, an upstream structural gene of the anthocyanin synthesis pathway, was only associated with the production of delphinidin-3-O-sambubioside. Compared to cultivar R, six DEGs were closely related to the three DEMs in cultivar S. Among these, the synthesis and transport of cyanidin-3-O-glucoside involved in *CHS1*, *CHS*, *UFGT1*, *CYP75A1*, *At4g26220*, and *HST.* The metabolite cyanidin-3-O-sophoroside is synthesized by three genes: *CHS1*, *CHS*, and *UFGT1*. In cultivar G, the gene with the highest expression level was *CHI*, an upstream structural gene involved in anthocyanin synthesis. *UFGT*, a downstream structural gene in anthocyanin synthesis, showed the highest expression level in cultivar R. The expression of *CHI* in cultivar G was approximately five times and two times that in R and S, respectively. *UFGT* gene expression in cultivar R was approximately 300 times and nine times higher than that in G and S, respectively.

### 2.8. QRT-PCR Validation for Candidate Genes

**Figure 8:**
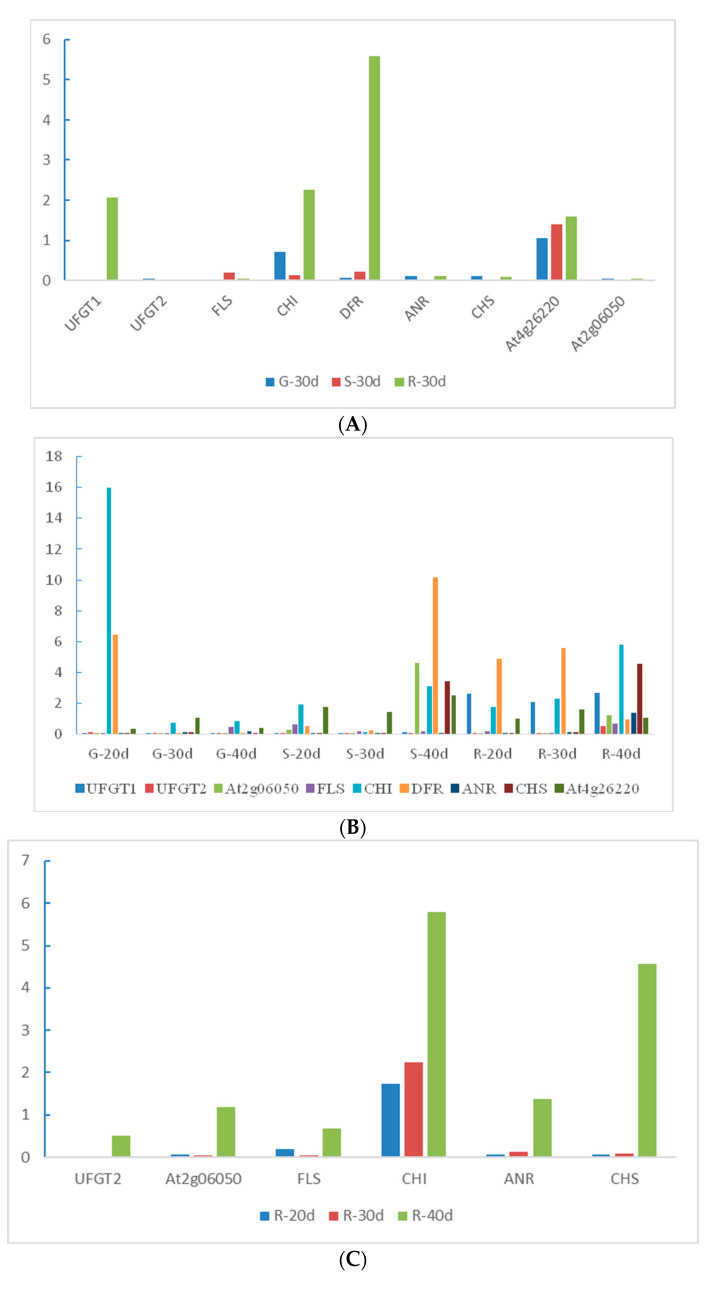
Expression patterns of nine genes obtained from RT-qPCR experiments. Relative expression values, normalized to NADPH, are shown as 2^−ΔΔCt^ relative to the mean expression levels in roselle calyxes. (**A**) Relative gene expression of nine candidate genes in different cultivars at 30 days after flowering (DAF). (**B**) Relative expression levels of nine candidate genes determined using qRT-PCR. (**C**) Relative expression of genes negatively related to anthocyanin synthesis at 20–40 DAF in the R cultivar. R, FZ-72 (red calyx); G, Baitao K (green calyx); S, MG5 (stripped calyx).

Based on the relationship between genes and metabolites as well as the expression levels of genes and metabolite contents, we identified nine genes related to anthocyanin synthesis, and qRT-PCR was used to detect their relative expression level in the calyxes at 30 d after flowering (Figure 8A). These nine genes belonged to the anthocyanin and flavonoid synthesis pathways; of these, one (Unigene00028712) belonged to the *CHS* family, one (Unigene00008117) belonged to the *CHI* family, two (Unigene00030931 and Unigene00088077) belonged to the *UFGT* family, one (Unigene00008034) belonged to the *FLS* family, one (Unigene00024761) belonged to the *ANR* family, and one (Unigene00008836) belonged to the *DFR* gene family. The other two (Unigene00007945 and Unigene00045492) belonged to the At2g06050 and At4g26220 families, respectively. qRT-PCR analysis of candidate genes showed that the gene expression trend was consistent with the transcriptome data (Figure 8A).

To determine the specific periods of gene expression that control anthocyanin synthesis, we performed qRT-PCR analysis on the three cultivars at different periods and determined their gene expression trends (Figure 8B). The highly expressed genes in the G cultivar were negatively correlated with anthocyanin content (absolute correlation coefficient > 0.8); these include *CHI*, *FLS*, *ANR*, *CHS,* and *UFGT2*. The highly expressed genes in R were strongly positively correlated with anthocyanin content (absolute correlation coefficient > 0.8), including *DFR* and *UFGT1*. In cultivar R, genes that showed a strongly negative association with anthocyanin synthesis gradually increased from day 30 to day 40 (Figure 7C). The other negatively regulated genes showed a trend of first decreasing and then increasing in cultivar R, with a turning point of 30 DAF. We speculated that this phenomenon may be related to the accumulation mechanism of flavonoids (rutin, quercetin-3-O-glucoside), and that the anthocyanin synthesis rate was faster in cultivar R at 30 DAF. Among the nine candidate genes verified in cultivar R at three different periods, only one gene (*DFR*) showed a gradual increase in expression with flowering time. In the combined analysis of 108 anthocyanins, *DFR* alone showed a strongly positive correlation with pelargonidin-3-O-glucoside and a negative correlation with rutin and quercetin-3-O-glucoside. The other *UFGT1* gene was found to have the highest expression in the R cultivar in transcriptome analysis. However, in the three periods of the R cultivar, gene expression levels gradually decreased from 20 to 40 DAF. Interestingly, *UFGT1* is involved in anthocyanin and flavonoid synthesis (rutin, quercetin-3-O-glucoside) in roselle. This gene showed opposite trends compared to those of *FLS*, *DFR*, *UFGT2*, and *CHS*, and the other genes involved in the synthesis of these two substances. In addition to conventional structural genes, *CHI*, *FLS*, *ANR*, *DFR*, *UFGT,* and *CCoAOMT* play essential roles in the anthocyanin synthesis pathway in roselle.

### 2.9. Essential Genes Responsible for Anthocyanin Synthesis in Roselle Calyxes

**Figure 9:**
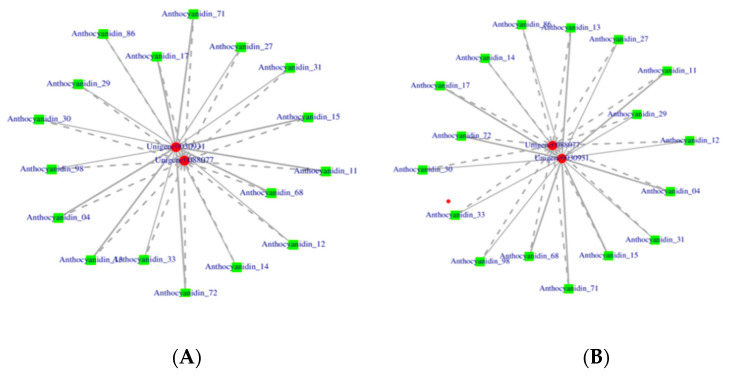
(**A**) *UFGT* gene and metabolite correlation network diagram of G vs. R. (**B**) *UFGT* gene and metabolite correlation network diagram of G vs. S. Solid lines indicate positive correlations whereas dashed lines indicate negative correlations. R, FZ-72 (red calyx); G, Baitao K (green calyx); S, MG5 (stripped calyx).

In the joint analysis, only two *UFGT* genes were found to be significantly different in the anthocyanin pathway, and these were analyzed further based on the relative expression level. The two *UFGT* showed contrasting expression in the two cultivars with apparent differences. The average FPKM value of *UFGT2* (unigene0088077) was 46.07 in cultivar G and 3.37 in cultivar R. *UFGT1* (unigen0030931) had an average FPKM value of 0.72 in cultivar G and 224.27 in cultivar R. *UFGT1* and *UFGT2* are involved in the synthesis of 17 kinds of anthocyanins and show opposite effects on the synthesis of unified anthocyanins (Figure 9). Compared with the other cultivars, *UFGT2* expression in cultivar G was the highest at the same stage. However, the relative expression level of *UFGT1* was opposite to that of *UFGT2*, and was the highest in cultivar R. We predicted that these two genes have antagonistic effects on the synthesis of roselle anthocyanins; however, this requires further functional verification.

**Figure 10:**
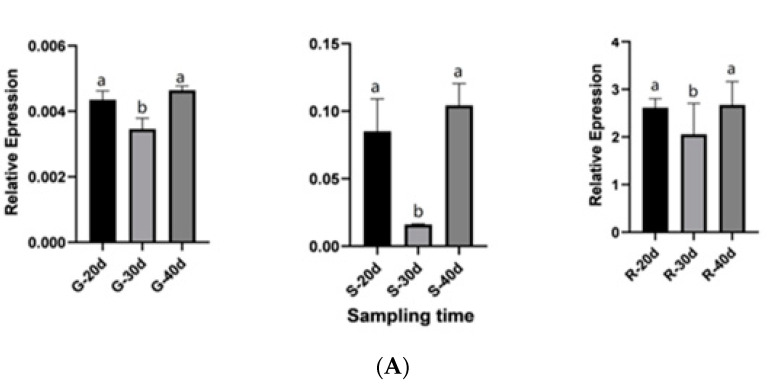

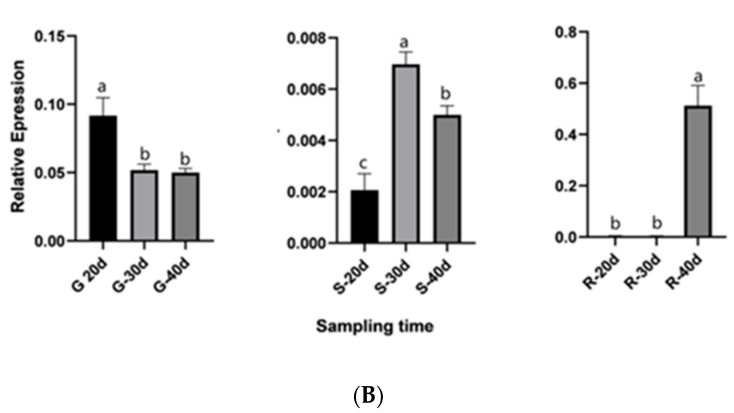
Relative expression levels of *UFGT1* and *UFGT2* genes in the same cultivars at different periods. (**A**) Relative expression of *UFGT1* in different cultivars. (**B**) Relative expression of *UFGT2* in different cultivars. Bars marked with the same and different letters indicate non-significant and significant differences, respectively. The significance level is α = 0.05. R, FZ-72 (red calyx); G, Baitao K (green calyx); S, MG5 (stripped calyx).

**Figure 11:**
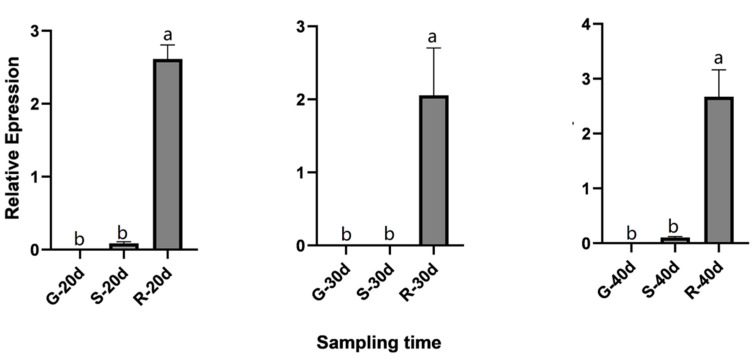
Relative expression levels of *UFGT1* genes at the same stage in different cultivars. R, FZ-72 (red calyx); G, Baitao K (green calyx); S, MG5 (stripped calyx). Bars marked with the same and different letters indicate non-significant and significant differences, respectively. The significance level is α = 0.05.

Interestingly, *UFGT1* expression gradually decreased in cultivars G and R with roselle maturity from 20 to 30 DAF (Figure 10). However, in cultivar S, *UFGT1* expression decreased sharply–from 20–30 DAF. The expression levels were restored in the three cultivars at 40 DAF. Further, the expression trends of *UFGT2* among the three cultivars and periods were inconsistent. In cultivar G, the expression level initially decreased and then increased. In cultivar S, it initially increased and then decreased. In cultivar R, the expression level declined gradually with flowering time. Comparison between different cultivars at the same stage showed that the expression level of *UFGT1* was relatively low in cultivar G (Figure 11), whereas it was highly expressed in cultivar R. However, the relative expression levels of *UFGT2* in different cultivars at the same stage revealed that the expression level of this gene in cultivar G was significantly higher than that in the other two cultivars.

## 3. Discussion

### 3.1. Anthocyanin Types and Content Identified in the Calyxes of Roselle

We examined 108 anthocyanins in three different roselle cultivars and detected six common anthocyanidins and 26 anthocyanins (nine types of cyanidins, seven types of delphinidins, four types of malvidins, two types of pelargonidins, three types of peonidins, and one type of petunidin). Delphinidin-3-o-glucoside and cyanidin-3-O-sambubioside were the two most abundant anthocyanins in the R and S cultivars, respectively. Jeny et al. reported two anthocyanidins (cyanidin and delphinidin) and four anthocyanins (cyanidin 3-O-glucoside, cyanidin 3-sambubioside, delphinidin 3-O-glucoside, and delphinidin 3-sambubioside) in roselle calyxes [26]. This difference in anthocyanin types may be attributed to differences in the cultivars and extraction technology used. However, cyanidin 3-sambubioside and delphinidin 3-sambubioside were identified to constitute the highest proportion of anthocyanins in the roselle calyx, consistent with our results. In a study by Trivellini [27], a reduction in cyanidin 3-sophoroside under salt stress resulted in a lighter calyx color, confirming that cyanidin is an essential component of calyx anthocyanins in roselle. In this study, metabolome analysis showed that the total anthocyanin content in cultivar R was the highest, followed by that in cultivar S, whereas cultivar G had the lowest content. This result is consistent with the phenotypic data, and indicates that the anthocyanin content can be preliminarily determined from the calyx color of roselle. These results were similar to those of Sirhan [24], showing that the anthocyanin content of p-J-O (purple, large-sized (jumbo), and opened calyx) was the highest, followed by that of PK-J-O (pink, large-sized (jumbo), and opened calyx), whereas the anthocyanin content of WT (White calyx) was the lowest, both in the main calyx and epicalyx of *Roselle solanum*. Among 108 metabolites detected in the G cultivar, rutin and quercetin-3-O-glucoside, belonging to flavonoids, had the highest content. According to Song et al. [28], flavonoids and flavonols can turn the color from yellow to white. Therefore, we inferred that rutin and quercetin-3-O-glucoside are involved in color development in the calyx of the G cultivar.

Based on phenotypic data, we considered that the content and type of anthocyanins in the G cultivar should be far less than those in the S cultivar. However, metabolome analysis indicated that many anthocyanins in the G cultivar are significantly upregulated compared with those in the S cultivar, possibly because of anthocyanin synthesis and accumulation of complex regulatory mechanisms. For example, the anthocyanin content in the roselle calyx is reported to be affected by hormone levels [29]. Further research is thus needed to understand the synthesis of anthocyanins and their accumulation mechanism in roselle.

### 3.2. There Were Several Essential Genes Controlling Anthocyanin Synthesis in Roselle Calyx

Interestingly, transcriptome analysis of *CHI* genes critical for anthocyanin synthesis, in the three cultivars, the highest expression in the G but not the R cultivar. As a critical upstream gene for the anthocyanin synthesis pathway, *CHI* is highly active in early stage, and its expression gradually decreases with maturity in the R cultivar. This is similar to the color-rendering mechanism of red jujube peels studied by Zhang [30]. They found that most structural genes in the flavonoid biosynthesis pathway are highly active in the white stage of the jujube, and are downregulated in the mature stage; further, some of these are completely silenced in the red cultivars. However, the *CHI* gene in roselle showed a significant downward trend at 20–30 DAF, whereas its expression gradually increased at 40 DAF. Studies have further shown that *HST*, a critical gene in PQ9 synthesis, is involved in the synthesis of PQ as well ascarotenoids, GA, and ABA [31]. *HST* is responsible for the development of flower organs and morphology [32]. However, *HST* inhibits anthocyanin synthesis in roselle. The *CCoAOMT* gene family is essential for lignin synthesis; in roselle, it has a strong inhibitory effect on anthocyanin synthesis in roselle [33]. *SAT* is reported to play a crucial role in cysteine and methionine synthesis [34] and may also be involved in the synthesis pathway of delphiniums, which exists with the synthesis pathway of anthocyanins by roselle. However, the expression level of this gene increased gradually with maturity in R and S cultivars; it first decreased and then increased in R cultivars. We speculate that these are minor genes involved in roselle anthocyanin synthesis, and their functions need to be verified further.

Anthocyanins are involved in complex biological pathways. So far, only the *CHS* and *F3H* genes have been cloned and analyzed by homologous cloning. *CHS* is a critical gene involved in anthocyanin synthesis in roselle [35]. In our transcriptome data, we found that the CHS content was highest in the R cultivar. In contrast, the qRT-PCR data showed that the *CHS* gene expression level gradually decreased with maturity in the R cultivar. In the S cultivar, the *CHS* expression level gradually increased. In the R cultivar, *CHS* expression decreased from 20 to 30 DFA and 30 to 40 DFA. *CHS* gene is thus an essential gene in anthocyanin synthesis. Further, *UFGT*, a key downstream gene in anthocyanin synthesis, has been reported in many species, including rose [36], mulberry [37], and rice [38]. In this study, two *UFGT* genes with opposite roles were screened, possibly because GTs can be divided into 91 distinct families [39] and different *UFGTs* perform different main functions. For instance, *UGT73C5* overexpression can improve resistance in *Arabidopsis* [40].

Genes involved in anthocyanin synthesis mainly include the upstream structural genes *CHS*, *CHI*, and *F3H*. The downstream structural genes *DFR*, *ANS*, and *UFGT*, interact with the transcription factors MYB, bHLH, and WD40 to form a complex protein MBW, which binds to the promoter of the anthocyanin glycoside synthesis gene and activates the target gene expression [41]. Currently, the mechanism of anthocyanin production in roselle sepals is unclear. We hypothesized that nine genes, including *CHI*, *CHS*, *FLS*, *ANR*, *DFR*, *UFGT*, *SAT*, *HST*, and *CCoAOMT,* are involved in the anthocyanin synthesis pathway in roselle calyxes. The functions of some genes in these gene families are not only associated with anthocyanin synthesis, but also involved in some other aspects, including stress response. The roles of these genes need be identified furtherly in the future.

### 3.3. Integrated Analysis of the Transcriptome and Metabolome

Metabolites are important indicators for identifying plant species and the physicochemical properties of plants, with approximately 200,000 to 1 million metabolite types [42]. In total, 41 metabolites were detected in roselle. A large number of DEGs were obtained using transcriptome data; these were correlated and analyzed with the differential metabolites in the metabolome. The internal changes of the organisms were analyzed at these two levels of cause and effect; key genes, metabolites, and metabolic pathways were locked, and core regulatory networks were constructed. The complex mechanisms of roselle anthocyanin synthesis were systematically and comprehensively analyzed.

Integrated analysis of the transcriptome and metabolome revealed only two *UFGT* genes in the anthocyanin synthesis pathway (ko00942), which showed contrasting regulatory relationships with metabolites. The expression of *UFGT1* in variety R was more than 300 times that in variety G. However, the expression of *UFGT2* was the lowest in variety R and the highest in variety G. The expression of *UFGT1* was directly proportional to anthocyanin content. The results of this study thus provide important clues for further research on the relationships between genes and metabolites.

## 4. Materials and Methods

### 4.1. Plant Materials

Three roselle cultivars were used: FZ-72, Baitao K, and MG5 with red, green, and stripped calyxes, respectively (Figure 1). We collected three cultivars from Yangzhong Experimental Station of Fujian Agricultural and Forestry University (118°21′–118°40′ E, 26°10′–26°22′ N), China. The calyxes of these three cultivars at 20, 30, and 40 days after flowering (DAFs) were harvested using a sharp blade, with three biological replicates. All samples were stored at −80 °C for further metabolomic, transcriptomic, and gene expression analyses.

### 4.2. Analysis of Anthocyanins in Roselle Using UPLC-Q-TOF/MS

Nine samples were collected from the calyxes of the three roselle cultivars, and three biological replicates were analyzed by liquid chromatography–tandem mass spectrometry (LC-MS/MS). The biological samples were vacuum-dried using a vacuum freeze-dryer (Scientz-100F) and crushed using a mixer mill (MM 400, Retsch) with zirconia beads for 1.5 min at 30 Hz [43]. Then, 50 mg of the resulting powder was dissolved in 500 μL extraction solution (50% methanol aqueous solution, containing 0.1% hydrochloric acid), vortexed for 5 min, ultrasonicated for 5 min, and centrifuged for 3 min (12,000 rpm, 4 °C); the supernatant was then absorbed. The operation was repeated once, and the samples were filtered through a microporous membrane (SCAA-104, 0.22 μm pore size; ANPEL, Shanghai, China, http://www.anpel.com.cn) (accessed on 20 November 2021) and stored in a vial for LC-MS/MS analysis. Anthocyanins were analyzed using a UPLC-ESI-MS/MS system (UPLC, SHIMADZU Nexera X2, www.shimadzu.com.cn/) (accessed on 25 November 2021); MS, Applied Biosystems 4500 Q TRAP, www.appliedbiosystems.com.cn/) (accessed on 25 November 2021). The data acquisition instrument system included UPLC (ExionLC™ AD, https://sciex.com.cn/) (accessed on 27 November 2021) and tandem mass spectrometry [44].

### 4.3. Metabolite Analysis

The mass spectrometry detection data of the metabolites in the experimental materials were based on the standard substances used to build the MWDB (Metware Biotech, Wuhan, China) database for qualitative analyses. The mass spectrum data were processed using Analyst 1.6.3 software. Multi Quant 3.0.3 software was used as a reference standard to conduct the necessary correction of mass spectrum peaks detected in different roselle samples to ensure the accuracy of qualitative and quantitative analysis (Supplement Figure S7 and Figure S8) [45]. The standard curve and equation were used to calculate the content of the substance in the actual samples [46]. The experimental results are categorized and presented as bar graphs and tables. Using UPLC-Q-TOF-MS, we determined the anthocyanin content and types in different roselle cultivars. The primary differential metabolites are shown in Table 1, and the differences in the metabolites of different cultivars are presented using a histogram.

The anthocyanin content data of different roselle samples were normalized by unit variance scaling, and R software was used for PCA, partial least squares discrimination analysis (OPLS-DA), and hierarchical cluster analysis for anthocyanin accumulation patterns among different roselle samples. R script was used to draw a clustering heat map for the standardized roselle data.

### 4.4. Screening of Differential Metabolites

By extracting and classifying calyx anthocyanins from three cultivars at 30 days after flowering, the mass spectrometry detection data were quantitatively analyzed to determine content differences between different substances and samples; a histogram was drawn according to these data. Finally, the differential metabolites and types of anthocyanins responsible for the color difference of the calyxes were determined.

Metabolites with a fold change ≥2 and ≤0.5 were selected as metabolites with a significant difference. The metabolites that ranked first and met the VIP screening conditions were displayed by comparing multiple differential changes in the quantitative metabolite information among the groups. The relationship between the differential metabolites in each group is displayed using a Venn diagram.

Differential metabolites that interacted with each other were used to form different metabolic pathways in roselle. We analyzed the metabolites using the KEGG database with a *p*-value < 0.01 in colored roselles among the three different cultivars. The differential metabolites were annotated and displayed using the KEGG database. The bubble diagram shows the degree of enrichment and the number of metabolites with a significant difference. All data and images were processed using GraphPad Prism v6.01 [47].

### 4.5. RNA Extraction and Illumina Sequencing

Total RNA was extracted from roselle calyxes using a Quick RNA isolation kit (ZH120; Hua Yueyang, China), and agarose gel electrophoresis was performed to analyze the integrity of the RNA samples. RNA purity was then determined using a NanoPhotometer. RNA integrity was measured using an Agilent 2100 BioAnalyzer, and RNA concentration was accurately quantified using a Qubit 2.0 Fluorometer. Approximately 200 bp cDNAs were screened using AMPure XP Beads for PCR amplification, and the PCR products were further purified using AMPure XP Beads to complete sequencing library construction. The constructed library was sequenced on the Illumina HiSeq^TM^2500 platform. Finally, the assembly integrity was evaluated using BUSCO (http://busco.ezlab.org/) (accessed on 12 November 2021).

### 4.6. RNA-Seq Analysis of Roselle Calyxes from Three Cultivars

To ensure data quality, we used fastp [48] to quality control the raw reads and obtain clean reads. Reads were assembled using Trinity software [49]. When a transcriptome was assembled using N50, the number of genes, and the values of Q20 to roughly evaluate the result of assembly, all unigenes were sorted from the longest to shortest, and the lengths were summed. When the accumulated fragment length reached 50% of the total fragment length (the length of all unigenes), the length and quantity of the corresponding fragments were considered the length and quantity of Unigene N50. However, these values cannot be used as standards for direct evaluation; therefore, assembly integrity must be evaluated using BUSCO [50]. The assembly quality was evaluated based on the unigene length. Transcriptome data were used to assemble transcripts, and multiple transcripts with variable splicing were clustered into a gene to form a unigene library. Gene expression levels were determined using the RPKM (reads per kb per million reads) method. Differential gene expression analyses between sample groups were performed using DESeq2 software [51]. The criteria for screening genes with significant differences were FDR < 0.05 and |log2FC| > 1.

According to the mapped read distribution of unigenes, fragment randomness, gene saturation, and nucleic acid sequences of roselle unigenes were compared using the BLAST, NT, NR, Swiss-Prot, and Pfam databases (evalue < 0.00001), and the protein with the highest sequence similarity of unigenes was obtained for the functional annotation of this unigene. After the unigene sequences were compared against the NR database, the sequences with the best results (the lowest E value) for each unigene were selected as the corresponding homologous sequences (the first sequence was taken in case of parallel results) to determine the species to which the homologous sequences belonged. The number of homologous sequences for each species was compared statistically. The central metabolic pathways and KEGG orthology were determined using KEGG annotation.

The assembled unigene sequences were compared with the protein databases NR, SwissProt, KEGG, and COG/KOG; the protein with the highest sequence similarity to the given unigene was used to obtain the functional annotation information for the unigenes. Based on the expression information of roselle genes, R (http://www.r-project.org/) (accessed on 20 November 2021) was used to perform principal component analysis (PCA) to determine the repeatability between samples and to help exclude outlier samples.

### 4.7. Conjoint Analysis

First, PCA was performed on the transcriptome and metabolome separately to determine differences between samples. Furthermore, according to the results of differential metabolite and differential gene analyses, the pathway enriched in the transcriptome and metabolome was used to draw a bubble map. The quantitative values of genes and metabolites in roselle were used for correlation analysis (the correlation coefficient was considered significant at more than 0.80, with a *p*-value less than 0.05), and a nine-image graph was used to analyze the expression trends. Moreover, a cluster heat map of the correlation coefficient was drawn, and a network diagram was used to visually express the correlation between metabolites and genes. The differential genes obtained using transcriptomic and metabolomic analyses were annotated using KEGG pathways, and the two omics datasets were verified to identify the metabolic pathways associated with crucial changes. Simultaneously, the association between the gene network and the phenotype of interest, as well as the core genes in the network, was explored, and the relationship between the genes controlling anthocyanin synthesis and calyx color was determined.

### 4.8. Protein Domain Prediction

The Pfam Scan [52] program developed by Sanger was used to predict the protein sequence encoded by the unigenes; this was compared with the Pfam database to obtain annotation information related to the protein structure encoded by the unigenes. The SMART protein domain was predicted using the HMMER [53] profile hidden Markov models for biological sequence analysis (http://hmmer.org/ (accessed on 1 December 2021). After the protein sequences predicted by unigenes were obtained, they were compared against the SMART database to obtain annotation information related to unigene protein structure.

### 4.9. Transcription Factor (TF) Identification

To predict and identify the transcription factors and their families in roselle, we compared the predicted protein sequences with the corresponding TF database [54,55] using HMMSCAN. The transcription factor results were statistically analyzed using a histogram.

### 4.10. Real-Time PCR Analysis

Nine candidate unigenes involved in anthocyanin synthesis from three roselle cultivars were screened for qRT-PCR analysis. First, purified RNA was reverse-transcribed to first-strand cDNA using a cDNA Reverse Transcription Kit (TransScript One-step gDNA Removal and cDNA Synthesis SuperMix) [56] based on the manufacturer’s instructions. Primers for the candidate unigenes were designed using IDT (https://sq.idtdna.com/Primerquest/Home/Index) (accessed on 10 January 2022) and Primer3 (Primer3 Input). Before qRT-PCR, the presence of primer dimers and primer quality were determined using standard PCR. The specific steps of qRT-PCR were conducted using PerfectStart^TM^ Green qPCR SuperMix as per the manufacturer’s instructions on a C1000 Touch™ Thermal Cycler System (Bio-RAD). Three biological replicates were performed for each sampling period, and three technical replicates were performed for each qRT-PCR to ensure the reliability of experimental data. The relative expression levels of differential genes were calculated using the 2^−ΔΔ^ Cp method, and the housekeeping gene Actin7 was used as an internal reference.

## 5. Conclusions

In this study, genes related to anthocyanin synthesis and the types of anthocyanins in roselle calyxes were determined through a combined analysis of the transcriptome and metabolome. In total, 41 anthocyanins were detected using UPLC-Q-TOF/MS analysis, among which the proportion of delphinidin-3-O-glucoside and cyanidin-3-O-sambubioside was the highest. Second, using transcriptome analysis, nine critical genes related to anthocyanin synthesis in the roselle calyx were identified. Finally, specific anthocyanins controlled by different genes in different cultivars were identified through joint data analysis. Overall, this study provides a theoretical basis for roselle breeding and understanding the anthocyanin synthesis pathway.

## Figures and Tables

**Table 1:** Anthocyanin contents among different cultivars.

Compounds	Mean(Group G) (μg/g)	Mean(Group S) (μg/g)	Mean(Group R) (μg/g)	RT (min)
Cyanidin-3-O-glucoside	0.16	12.23	40.58	7.48
Cyanidin-3-O-(6-O-malonyl-beta-D-glucoside)	ND	ND	0.01	10.26
Cyanidin-3-O-sambubioside-5-O-glucoside	ND	1.27	5.72	5.66
Cyanidin-3-O-xyloside	ND	0.01	0.06	9.69
Cyanidin-3-O-rutinoside-5-O-glucoside	ND	0.01	0.01	5.51
Cyanidin-3-O-galactoside	ND	0.27	1.30	6.94
Cyanidin-3-O-arabinoside	ND	0.04	0.31	7.91
Cyanidin-3-O-sophoroside	ND	2.15	11.23	6.92
Cyanidin-3-O-rutinoside	ND	0.20	0.46	7.98
Cyanidin-3,5,3’-O-triglucoside	ND	0.03	0.11	5.18
Cyanidin-3-O-sambubioside	0.11	330.06	531.37	7.59
Cyanidin-3,5-O-diglucoside	ND	0.05	0.14	5.45
Delphinidin-3-O-rutinoside	0.03	2.19	3.53	7.08
Delphinidin-3-O-(6-O-malonyl-beta-D-glucoside)	0.08	ND	ND	9.56
Delphinidin-3-O-rutinoside-5-O-glucoside	0.16	0.06	ND	7.10
Delphinidin-3-O-sambubioside-5-O-glucoside	ND	1.36	2.32	4.30
Delphinidin-3-O-rhamnoside	ND	0.03	0.08	8.05
Delphinidin-3-O-glucoside	ND	35.37	70.47	6.48
Delphinidin-3-O-arabinoside	ND	0.07	0.17	7.07
Delphinidin-3-O-sambubioside	0.26	851.97	955.11	6.37
Delphinidin-3-O-sophoroside	ND	44.60	85.08	6.06
Malvidin-3-O-glucoside	0.01	ND	0.01	9.37
Pelargonidin-3-O-galactoside	ND	ND	0.15	7.77
Pelargonidin-3-O-sambubioside	ND	3.65	16.18	8.76
Pelargonidin-3-O-glucoside	ND	0.24	4.17	8.42
Pelargonidin-3-O-sophoroside	ND	0.01	0.06	7.71
Peonidin-3-O-sambubioside-5-O-glucoside	ND	ND	0.06	6.69
Peonidin-3,5-O-diglucoside	ND	ND	0.01	6.76
Peonidin-3-O-sambubioside	ND	0.36	1.38	9.00
Peonidin-3-O-glucoside	ND	0.08	0.11	9.02
Peonidin	0.02	0.07	0.03	9.76
Petunidin-3-O-galactoside	ND	2.84	7.90	8.10
Petunidin-3-O-glucoside	ND	0.58	1.62	8.11
Petunidin-3-O-arabinoside	ND	0.03	0.04	8.53
Petunidin-3,5-O-diglucoside	ND	ND	0.03	6.17
Procyanidin B3	0.07	0.26	0.23	3.45
Procyanidin B2	6.62	8.28	3.57	5.57
Procyanidin B1	0.64	0.82	1.17	3.86
Rutin	615.25	308.06	61.94	11.31
Quercetin-3-O-glucoside	312.22	120.67	49.38	11.30
Kaempferol-3-O-rutinoside	17.90	43.03	12.72	12.45

ND, not detected. R, FZ-72 (red calyx); G, Baitao K (green calyx); S, MG5 (stripped calyx).

## Data Availability

All data generated during this study are included in this published article and its supplementary information files, and the raw data used or analyzed during the current study are available from the NCBI (https://submit.ncbi.nlm.nih.gov/) with PRJNA885398.

## Supplementary Materials

The following supporting information can be downloaded at: https://www.mdpi.com/article/10.3390/ijms232213908/s1.
