# Supplementary material for: Novel Insights into Anthocyanin Synthesis in the Calyx of Roselle Using Integrated Transcriptomic and Metabolomic Analyses
Source: Int J Mol Sci. 2022 Nov 11;23(22):13908. doi: 10.3390/ijms232213908 (PMC9693987; doi:10.3390/ijms232213908)
Supplement: Supplementary file 1 [file ijms-23-13908-s001.zip › ijms-1971044-supplementary.pdf]

Supplement Table S1. List of 108 metabolites detected in roselle

| Index | Compounds | Class |
|---|---|---|
| Anthocyanidin_02 | Cyanidin-3-(6''-caffeylsophoroside)-5-glucoside | Cyanidin |
| Anthocyanidin_03 | Cyanidin-3,5,3'-O-triglucoside | Cyanidin |
| Anthocyanidin_04 | Cyanidin-3,5-O-diglucoside | Cyanidin |
| Anthocyanidin_05 | Cyanidin-3-O-(6-O-malonyl-beta-D-glucoside) | Cyanidin |
| Anthocyanidin_06 | Cyanidin-3-O-(6-O-p-coumaroyl)-glucoside | Cyanidin |
| Anthocyanidin_07 | Cyanidin-3-O-5-O-(6-O-coumaroyl)-diglucoside | Cyanidin |
| Anthocyanidin_08 | Cyanidin-3-O-arabinoside | Cyanidin |
| Anthocyanidin_09 | Cyanidin-3-O-(6''-ferulylsophoroside)-5-glucoside | Cyanidin |
| Anthocyanidin_10 | Cyanidin-3-O-galactoside | Cyanidin |
| Anthocyanidin_100 | Petunidin-3-O-sambubioside | Petunidin |
| Anthocyanidin_101 | Petunidin-3-O-sophoroside | Petunidin |
| Anthocyanidin_102 | Petunidin-3-O-sambubioside-5-O-glucoside | Petunidin |
| Anthocyanidin_103 | Procyanidin A1 | Procyanidin |
| Anthocyanidin_104 | Procyanidin A2 | Procyanidin |
| Anthocyanidin_105 | Procyanidin B1 | Procyanidin |
| Anthocyanidin_106 | Procyanidin B2 | Procyanidin |
| Anthocyanidin_107 | Procyanidin B3 | Procyanidin |
| Anthocyanidin_108 | Procyanidin C1 | Procyanidin |
| Anthocyanidin_11 | Cyanidin-3-O-glucoside | Cyanidin |
| Anthocyanidin_12 | Cyanidin-3-O-rutinoside | Cyanidin |
| Anthocyanidin_13 | Cyanidin-3-O-rutinoside-5-O-glucoside | Cyanidin |
| Anthocyanidin_14 | Cyanidin-3-O-sambubioside | Cyanidin |
| Anthocyanidin_15 | Cyanidin-3-O-sophoroside | Cyanidin |
| Anthocyanidin_16 | Cyanidin-3-O-xyloside | Cyanidin |
| Anthocyanidin_17 | Cyanidin-3-O-sambubioside-5-O-glucoside | Cyanidin |
| Anthocyanidin_18 | Delphinidin | Delphinidin |
| Anthocyanidin_19 | Delphinidin-3,5-O-diglucoside | Delphinidin |
| Anthocyanidin_20 | Delphinidin-3-O-(6-O-acetyl)-glucoside | Delphinidin |
| Anthocyanidin_21 | Delphinidin-3-O-(6-O-malonyl-beta-D-glucoside) | Delphinidin |
| Anthocyanidin_22 | Delphinidin-3-O-(6-O-malonyl)-glucoside-3'-glucoside | Delphinidin |
| Anthocyanidin_23 | Delphinidin-3-O-(6-O-p-coumaroyl)-glucoside | Delphinidin |
| Anthocyanidin_24 | Delphinidin-3-O-5-O-(6-O-coumaroyl)-diglucoside | Delphinidin |
| Anthocyanidin_25 | Delphinidin-3-O-arabinoside | Delphinidin |
| Anthocyanidin_26 | Delphinidin-3-O-galactoside | Delphinidin |
| Anthocyanidin_27 | Delphinidin-3-O-glucoside | Delphinidin |
| Anthocyanidin_28 | Delphinidin-3-O-rhamnoside | Delphinidin |
| Anthocyanidin_29 | Delphinidin-3-O-rutinoside | Delphinidin |
| Anthocyanidin_30 | Delphinidin-3-O-sambubioside | Delphinidin |
| Anthocyanidin_31 | Delphinidin-3-O-sophoroside | Delphinidin |
| Anthocyanidin_32 | Delphinidin-3-O-rutinoside-5-O-glucoside | Delphinidin |
| Anthocyanidin_33 | Delphinidin-3-O-sambubioside-5-O-glucoside | Delphinidin |

| | | |
|---|---|---|
| Anthocyanidin_34 | Afzelin | flavonoid |
| Anthocyanidin_35 | Chalcone | flavonoid |
| Anthocyanidin_36 | Dihydrokaempferol | flavonoid |
| Anthocyanidin_37 | Dihydromyricetin | flavonoid |
| Anthocyanidin_38 | Kaempferol-3-O-rutinoside | flavonoid |
| Anthocyanidin_39 | Naringenin | flavonoid |
| Anthocyanidin_40 | Naringenin-7-O-glucoside | flavonoid |
| Anthocyanidin_41 | Quercetin-3-O-glucoside | flavonoid |
| Anthocyanidin_42 | Rutin | flavonoid |
| Anthocyanidin_43 | Malvidin | Malvidin |
| Anthocyanidin_44 | Malvidin-3,5-O-diglucoside | Malvidin |
| Anthocyanidin_45 | Malvidin-3-O-(6"-acetylglucoside)-5-glucoside | Malvidin |
| Anthocyanidin_46 | Malvidin-3-O-(6-O-malonyl-beta-D-glucoside) | Malvidin |
| Anthocyanidin_47 | Malvidin-3-O-(6-O-p-coumaroyl)-glucoside | Malvidin |
| Anthocyanidin_48 | Malvidin-3-O-5-O-(6-O-coumaroyl)-diglucoside | Malvidin |
| Anthocyanidin_49 | Malvidin-3-O-arabinoside | Malvidin |
| Anthocyanidin_50 | Malvidin-3-O-galactoside | Malvidin |
| Anthocyanidin_51 | Malvidin-3-O-glucoside | Malvidin |
| Anthocyanidin_52 | Malvidin-3-O-rutinoside | Malvidin |
| Anthocyanidin_53 | Malvidin-3-O-sambubioside | Malvidin |
| Anthocyanidin_54 | Malvidin-3-O-sophoroside | Malvidin |
| Anthocyanidin_55 | Malvidin-3-O-sambubioside-5-O-glucoside | Malvidin |
| Anthocyanidin_56 | Pelargonidin | Pelargonidin |
| Anthocyanidin_57 | Pelargonidin-3-(6"-caffeylsophoroside)-5-glucoside | Pelargonidin |
| Anthocyanidin_58 | Pelargonidin-3-sophoroside-5-glucoside | Pelargonidin |
| Anthocyanidin_59 | Pelargonidin-3,5-O-diglucoside | Pelargonidin |
| Anthocyanidin_60 | Pelargonidin-3-O-(6"-ferulylsambubioside)-5-O-(malonyl)-glucoside | Pelargonidin |
| Anthocyanidin_61 | Pelargonidin-3-O-(6-O-malonyl-beta-D-glucoside) | Pelargonidin |
| Anthocyanidin_62 | Pelargonidin-3-O-(6-O-p-coumaroyl)-glucoside | Pelargonidin |
| Anthocyanidin_63 | Pelargonidin-3-O-[2-O-glucosyl-6-O-p-coumaroyl-glucoside]-5-O-glucoside | Pelargonidin |
| Anthocyanidin_64 | Pelargonidin-3-O-5-O-(6-O-coumaroyl)-diglucoside | Pelargonidin |
| Anthocyanidin_65 | Pelargonidin-3-O-[6-O-feruloyl-2-O-glucosyl-glucoside]-5-O-glucoside | Pelargonidin |
| Anthocyanidin_66 | Pelargonidin-3-O-arabinoside | Pelargonidin |
| Anthocyanidin_67 | Pelargonidin-3-O-galactoside | Pelargonidin |
| Anthocyanidin_68 | Pelargonidin-3-O-glucoside | Pelargonidin |
| Anthocyanidin_69 | Pelargonidin-3-O-rutinoside | Pelargonidin |
| Anthocyanidin_70 | Pelargonidin-3-O-rutinoside-5-O-glucoside | Pelargonidin |
| Anthocyanidin_71 | Pelargonidin-3-O-sambubioside | Pelargonidin |
| Anthocyanidin_72 | Pelargonidin-3-O-sophoroside | Pelargonidin |
| Anthocyanidin_73 | Pelargonidin-3-O-sophoroside-5-O-(malonyl)-glucoside | Pelargonidin |
| Anthocyanidin_74 | Pelargonidin-3-O-sambubioside-5-O-glucoside | Pelargonidin |
| Anthocyanidin_75 | Peonidin | Peonidin |
| Anthocyanidin_76 | Peonidin-3-(caffeoyl-glucosyl-glucoside)-5-glucoside | Peonidin |
| Anthocyanidin_77 | Peonidin-3-sophoroside-5-glucoside | Peonidin |

| | | |
|---|---|---|
| Anthocyanidin_78 | Peonidin-3,5-O-diglucoside | Peonidin |
| Anthocyanidin_79 | Peonidin-3-O-(6-O-malonyl-beta-D-glucoside) | Peonidin |
| Anthocyanidin_80 | Peonidin-3-O-(6-O-p-coumaroyl)-glucoside | Peonidin |
| Anthocyanidin_81 | Peonidin-3-O-5-O-(6-O-coumaroyl)-diglucoside | Peonidin |
| Anthocyanidin_82 | Peonidin-3-O-arabinoside | Peonidin |
| Anthocyanidin_83 | Peonidin-3-O-caffeoyl-feruloyl-sophoroside-5-glucoside | Peonidin |
| Anthocyanidin_84 | Peonidin-3-O-(6"-ferulylsophoroside)-5-glucoside | Peonidin |
| Anthocyanidin_85 | Peonidin-3-O-galactoside | Peonidin |
| Anthocyanidin_86 | Peonidin-3-O-glucoside | Peonidin |
| Anthocyanidin_87 | Peonidin-3-O-P-hydroxybenzoylsophoroside-5-glucoside | Peonidin |
| Anthocyanidin_88 | Peonidin-3-O-rutinoside | Peonidin |
| Anthocyanidin_89 | Peonidin-3-O-sambubioside | Peonidin |
| Anthocyanidin_90 | Peonidin-3-O-sophoroside | Peonidin |
| Anthocyanidin_91 | Peonidin-3-O-sambubioside-5-O-glucoside | Peonidin |
| Anthocyanidin_92 | Petunidin-3,5-O-diglucoside | Petunidin |
| Anthocyanidin_93 | Petunidin-3-O-(6-O-malonyl-beta-D-glucoside) | Petunidin |
| Anthocyanidin_94 | Petunidin-3-O-(6-O-p-coumaroyl)-glucoside | Petunidin |
| Anthocyanidin_95 | Petunidin-3-O-5-O-(6-O-coumaroyl)-diglucoside | Petunidin |
| Anthocyanidin_96 | Petunidin-3-O-arabinoside | Petunidin |
| Anthocyanidin_97 | Petunidin-3-O-galactoside | Petunidin |
| Anthocyanidin_98 | Petunidin-3-O-glucoside | Petunidin |
| Anthocyanidin_99 | Petunidin-3-O-rutinoside | Petunidin |

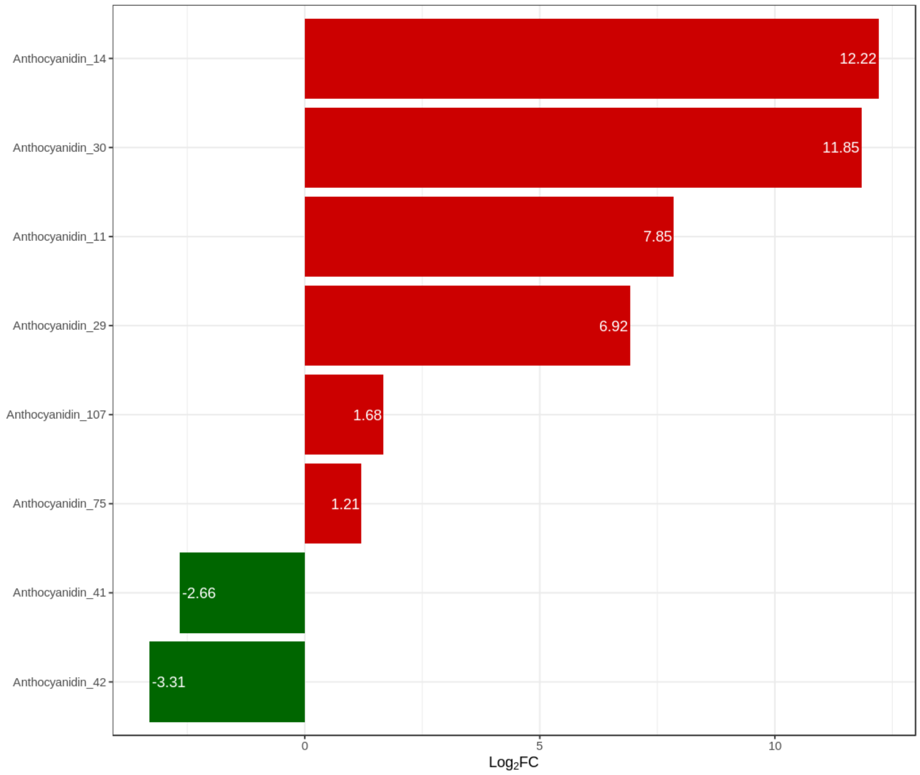
Anthocyanidin_14
Anthocyanidin_30
Anthocyanidin_11
Anthocyanidin_29
Anthocyanidin_107
Anthocyanidin_75
Anthocyanidin_41
Anthocyanidin_42
12.22
11.85
7.85
6.92
1.68
1.21
-2.66
-3.31
0
5
10
Log2FC

(**A**)

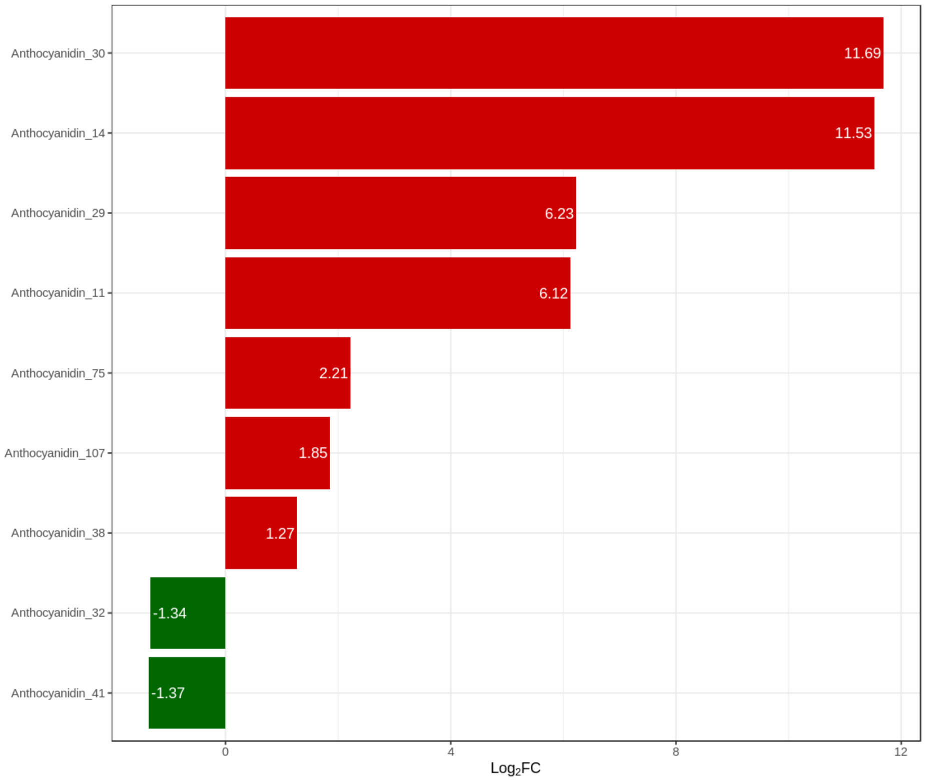
Anthocyanidin_30
Anthocyanidin_14
Anthocyanidin_29
Anthocyanidin_11
Anthocyanidin_75
Anthocyanidin_107
Anthocyanidin_38
Anthocyanidin_32
Anthocyanidin_41
11.69
11.53
6.23
6.12
2.21
1.85
1.27
-1.34
-1.37
0
4
8
12
Log2FC

(**B**)

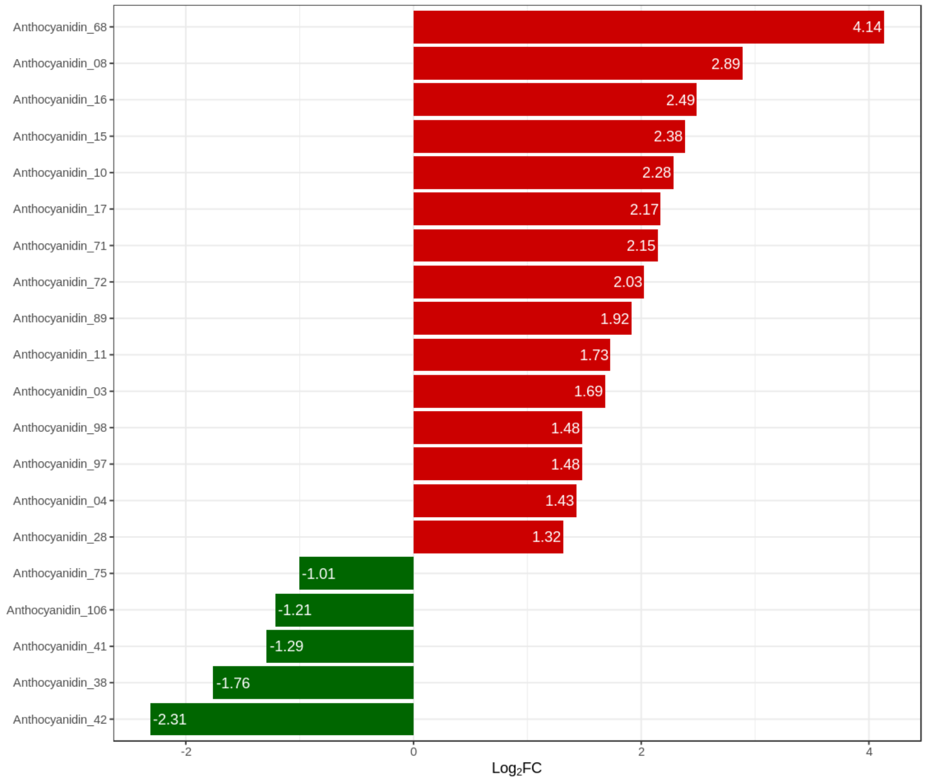


(**C**)

Supplement Figure S1. Differential metabolites bar chart. (**A**) Significantly different metabolite bar chart between G and R. (**B**) Significantly different metabolite bar chart between G and S. (**C**) Significantly different metabolite bar chart between S and R. R, FZ-72 (red calyx); G, Baitao K (green calyx); S, MG5 (stripped calyx)

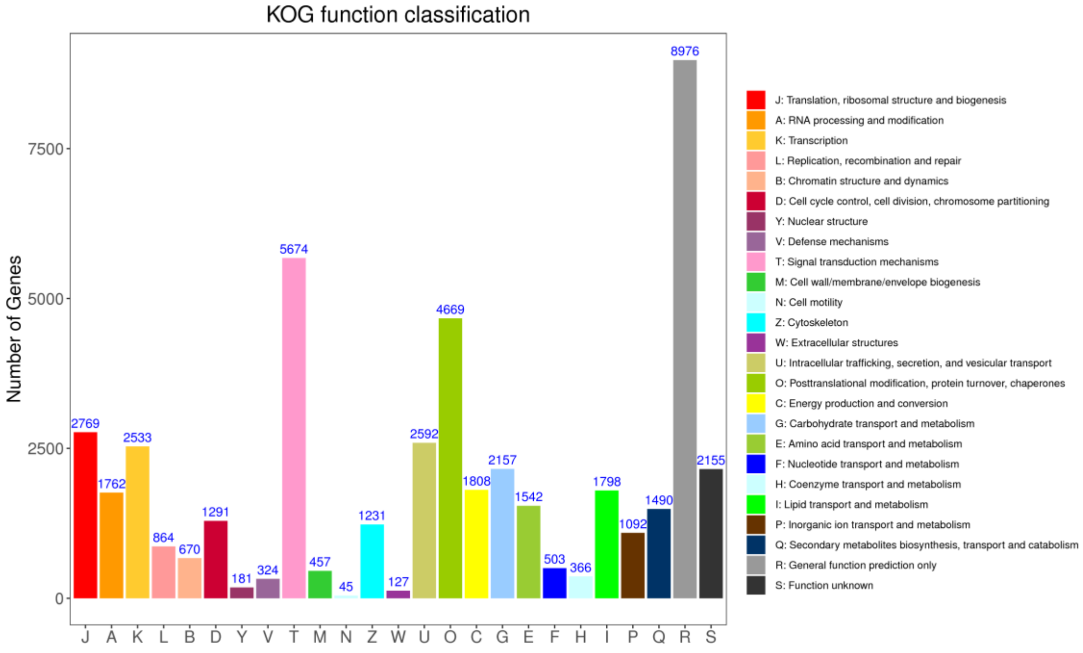


Supplement Figure S2. KOG functional classification statistics.

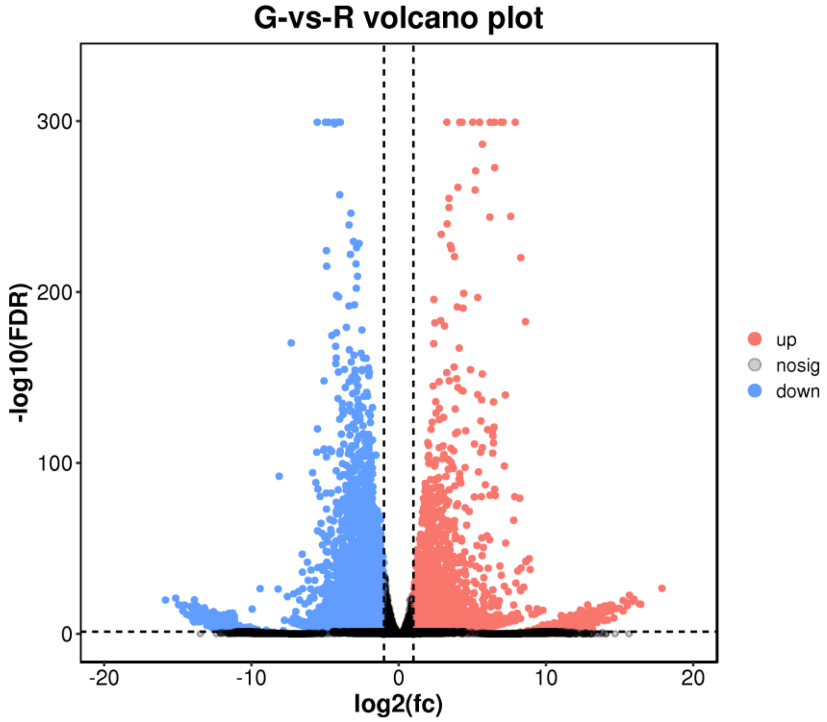

G-vs-R volcano plot
-log10(FDR)
300
200
100
0
-20
-10
0
10
20
log2(fc)
up
nosig
down


(**A**)

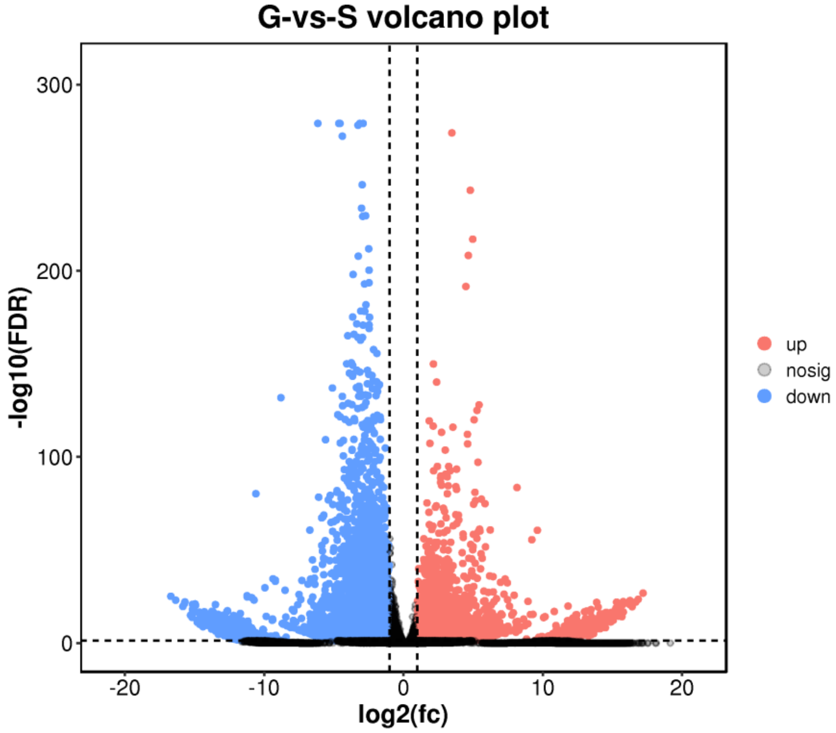

G-vs-S volcano plot
-log10(FDR)
300
200
100
0
-20
-10
0
10
20
log2(fc)
up
nosig
down


(**B**)

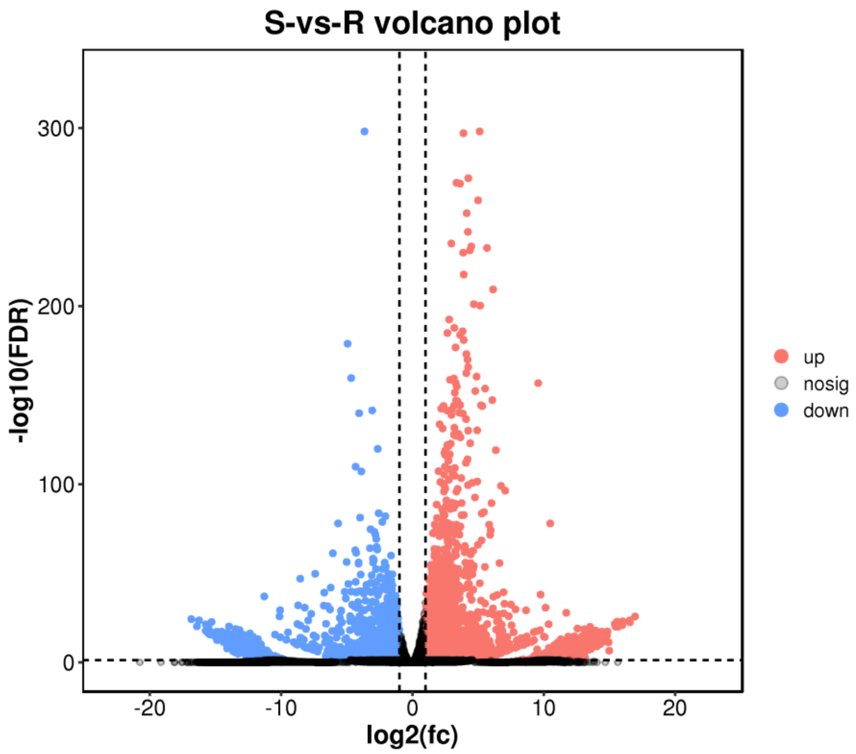


(C)

Supplement Figure S3. Volcano plots for different combinations. A larger absolute value of the abscissa indicates a greater difference between the two groups of genes; a larger value of the ordinate indicates a more significant difference in gene expression between groups and a more reliable result. R, FZ-72 (red calyx); G, Baitao K (green calyx); S, MG5 (stripped calyx)

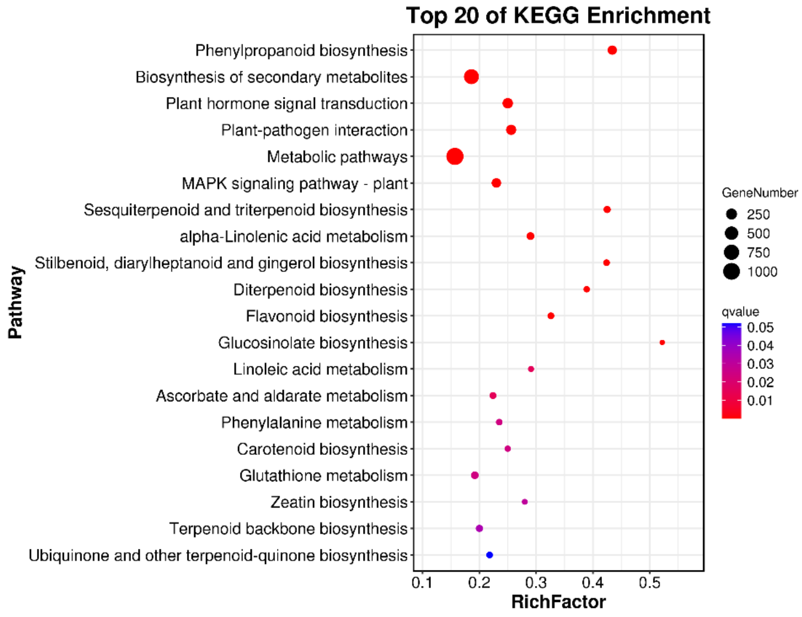


(A)

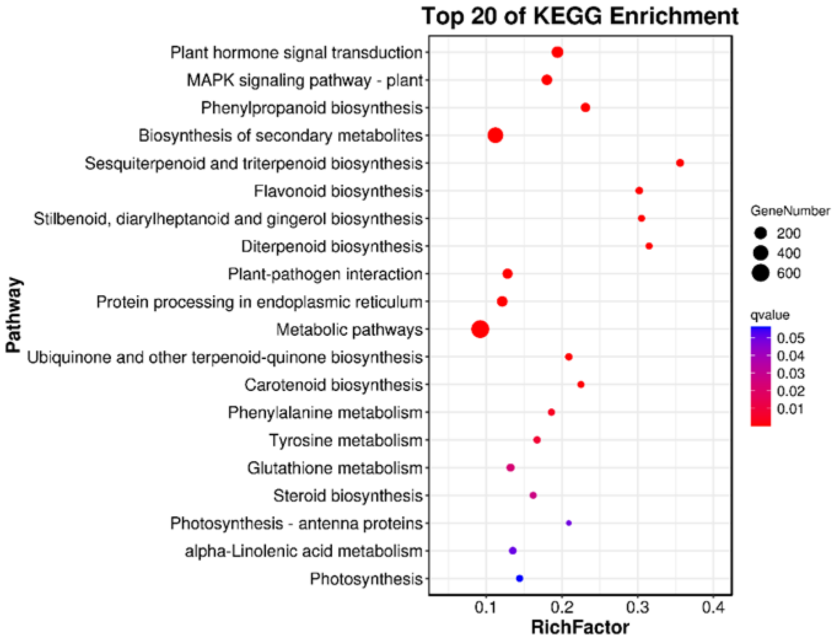


(**B**)

Supplement Figure S4. Kyoto Encyclopedia of Genes and Genomes (KEGG) enrichment bubble plot. (A) G*vs*S differential gene enrichment bubble plot. (B) S*vs*R differential gene enrichment bubble plot. The top 20 pathways with the smallest Q values were used to construct the graph. The ordinate indicates the pathway and the abscissa indicates the enrichment factor. The size indicates the quantity. The redder the color, the smaller is the Q value. R, FZ-72 (red calyx); G, Baitao K (green calyx); S, MG5 (stripped calyx)

Supplement Table S2. Differentially expressed genes (DEGs) in the flavonoid synthesis pathway (ko00941) and anthocyanin synthesis pathway (ko00942) among the three combinations.

| Unigene ID | short name | Full name |
|---|---|---|
| Unigene0029458 | / | |
| Unigene0009721 | ACT | adult cataract |
| Unigene0097172 | ACT | adult cataract |
| Unigene0018909 | AN3 | ANGUSTIFOLIA 3, |
| Unigene0024761 | ANR | anthocyanidin reductase |
| Unigene0009339 | ANS | anthocyanidin synthase |
| Unigene0045492 | At4g26220 | S-adenosyl-L-methionine-dependent methyltransferases superfamily protein |
| Unigene0014782 | CCOAOMT1 | S-adenosyl-L-methionine-dependent methyltransferases superfamily protein |
| Unigene0074946 | CCOAOMT1 | S-adenosyl-L-methionine-dependent methyltransferases superfamily protein |
| Unigene0006017 | CCOAOMT5 | caffeoyl-CoA O-methyltransferase 5 |
| Unigene0065186 | CCOAOMT5 | caffeoyl-CoA O-methyltransferase 6 |
| Unigene0074947 | CCOMT | catechol-O-methyltransferase |
| Unigene0008117 | CHI3 | chitinase |
| Unigene0026462 | CHS | chalcone synthase |
| Unigene0028712 | CHS | chalcone synthase |
| Unigene0076313 | CHS | chalcone synthase |

| | | |
|---|---|---|
| Unigene0007849 | CHS1 | chalcone synthase 1 |
| Unigene0022461 | CHS1 | chalcone synthase 1 |
| Unigene0053976 | CHS2 | chalcone synthase 2 |
| Unigene0053978 | CHS4 | chalcone synthase 4 |
| Unigene0009044 | CYP73A12 | |
| Unigene0000986 | CYP73A13 | |
| Unigene0037625 | CYP75A1 | |
| Unigene0009121 | CYP98A2 | cytochrome P450 98A2 |
| Unigene0043946 | CYP98A2 | cytochrome P450 98A3 |
| Unigene0008836 | DFR | dihydroflavonol 4-reductase |
| Unigene0095921 | DFR | dihydroflavonol 5-reductase |
| Unigene0008826 | FAOMT | anthocyanin O-methyltransferase |
| Unigene0008827 | FAOMT | anthocyanin O-methyltransferase |
| Unigene0008828 | FAOMT | anthocyanin O-methyltransferase |
| Unigene0008034 | FLS | flavonol synthase |
| Unigene0001791 | HST | homogentisate prenyltransferase |
| Unigene0008344 | HST | homogentisate prenyltransferase |
| Unigene0016835 | HST | homogentisate prenyltransferase |
| Unigene0026470 | HST | homogentisate prenyltransferase |
| Unigene0029457 | HST | homogentisate prenyltransferase |
| Unigene0035514 | HST | homogentisate prenyltransferase |
| Unigene0035515 | HST | homogentisate prenyltransferase |
| Unigene0045377 | HST | homogentisate prenyltransferase |
| Unigene0052944 | HST | homogentisate prenyltransferase |
| Unigene0080927 | HST | homogentisate prenyltransferase |
| Unigene0083370 | HST | homogentisate prenyltransferase |
| Unigene0101365 | HST | homogentisate prenyltransferase |
| Unigene0007848 | PKS5 | protein kinase5 |
| Unigene0007635 | SAT | serine acetyltransferase |
| Unigene0047199 | SAT | serine acetyltransferase |
| Unigene0105300 | SAT | serine acetyltransferase |
| Unigene0030931 | UFGT | anthocyanidin 3-O-glucosyltransferase 2 |
| Unigene0088077 | UFGT | anthocyanidin 3-O-glucosyltransferase 3 |

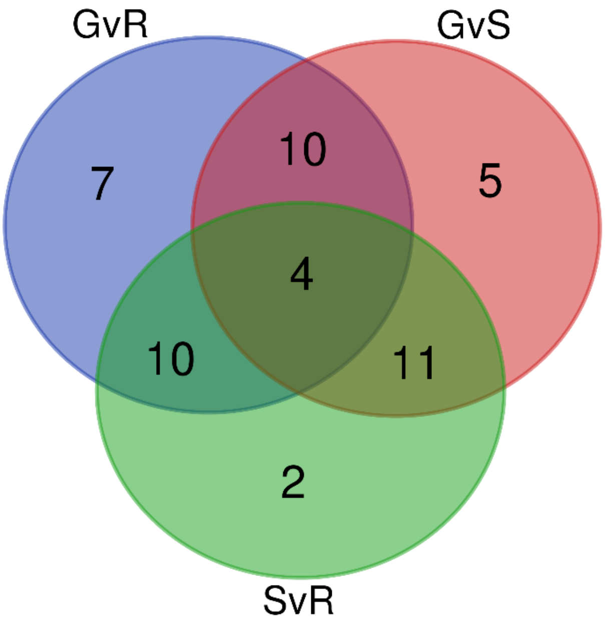


Supplement Figure S5. Venn diagram of significantly different genes in the flavonoid (ko00941) and anthocyanin (ko00942) synthesis pathways.

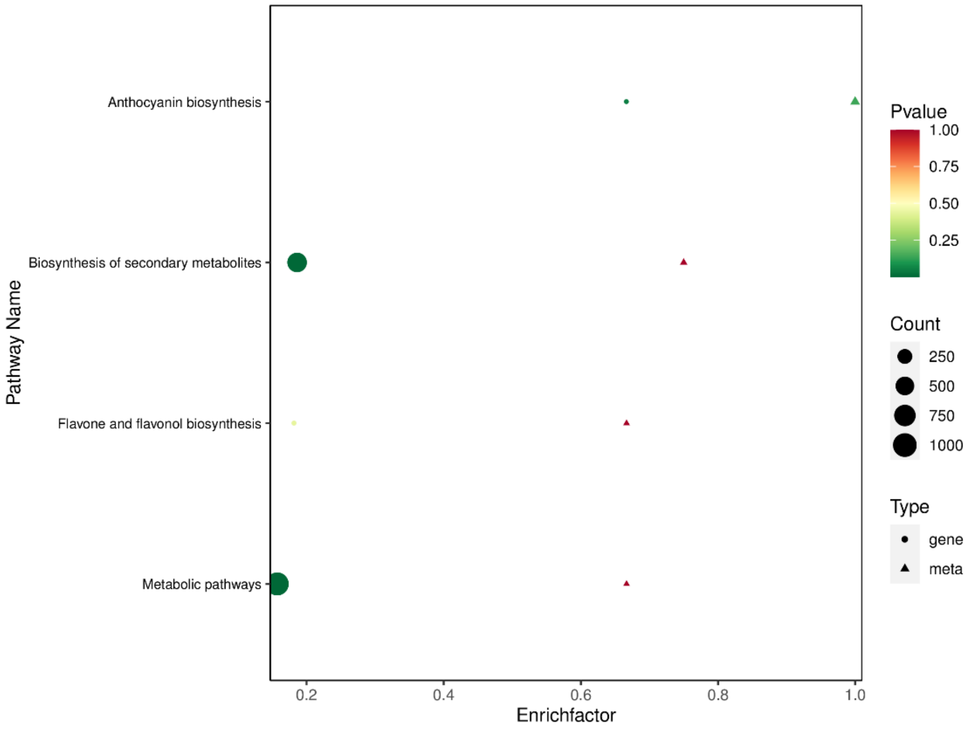


**(A)**

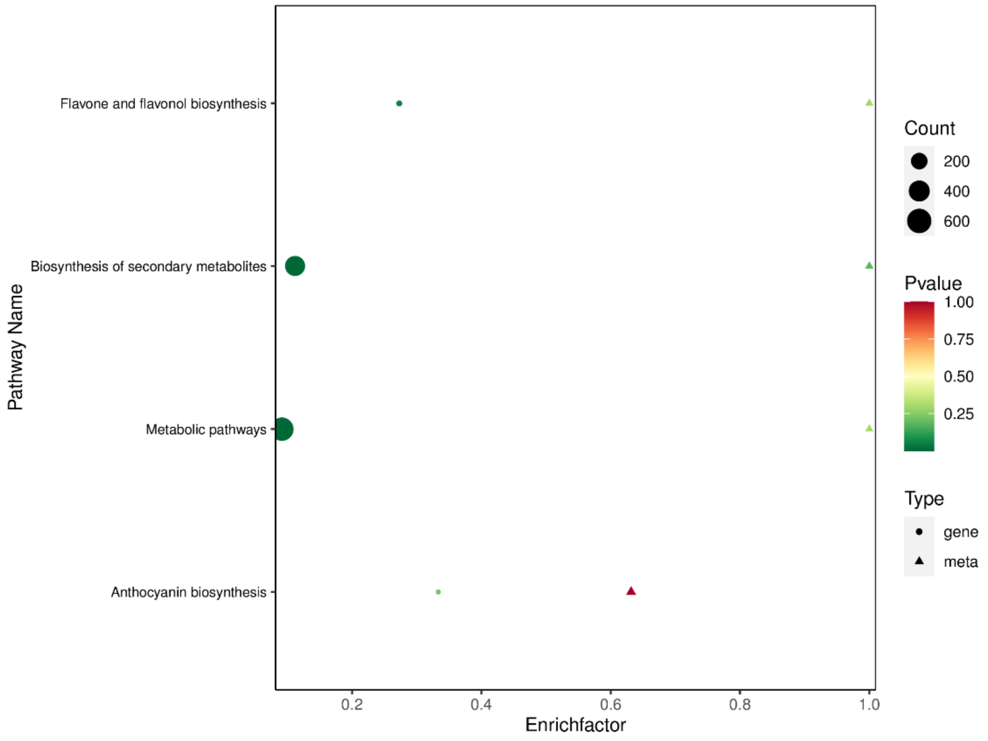


**(B)**

Supplement Figure S6. Bubble chart for the combined analysis of differentially expressed genes and differential metabolites. (A) Kyoto encyclopedia of genes and genomes (KEGG) enrichment bubble plot for G and S. (B KEGG) enrichment bubble plot for S and R. R, FZ-72 (red calyx); G, Baitao K (green calyx); S, MG5 (stripped calyx)

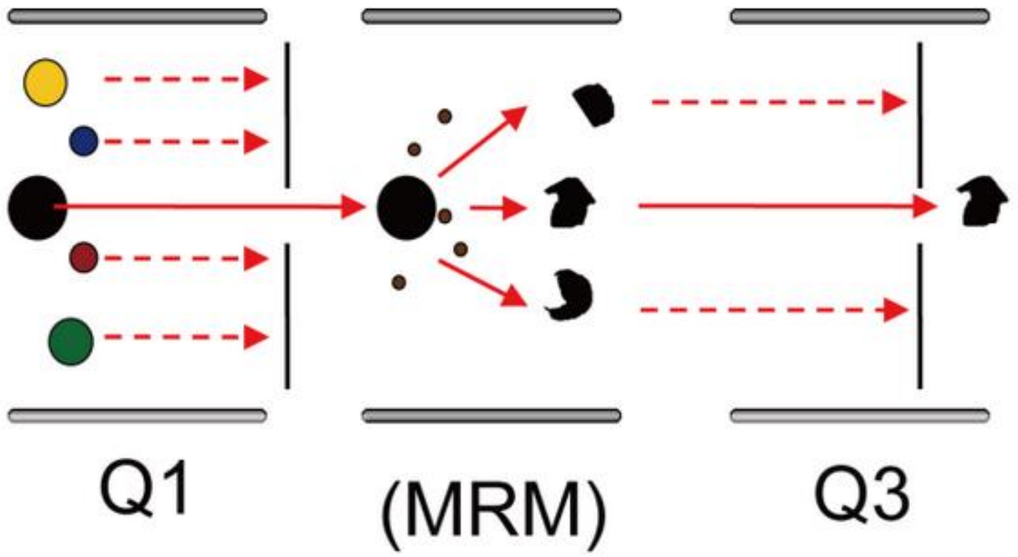


Supplement Figure S7. Multiple Reaction Monitoring (MRM) analysis of anthocyanin content in roselle calyx.

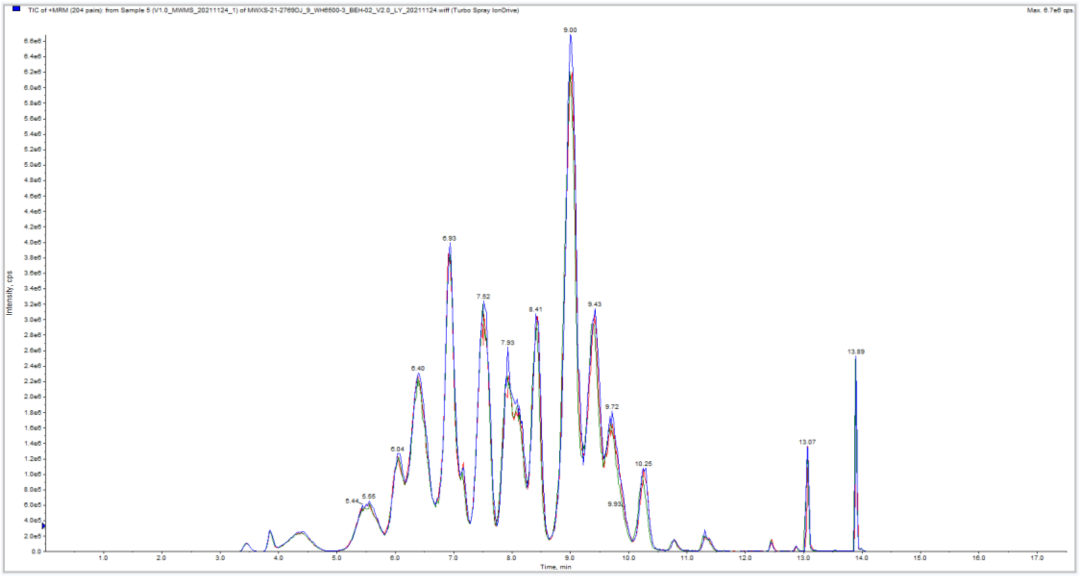


Supplement Figure S8. Chromatographic analysis of total ion flow.
